# Use cases, best practice and reporting standards for metabolomics in regulatory toxicology

**DOI:** 10.1038/s41467-019-10900-y

**Published:** 2019-07-10

**Authors:** Mark R. Viant, Timothy M. D. Ebbels, Richard D. Beger, Drew R. Ekman, David J. T. Epps, Hennicke Kamp, Pim E. G. Leonards, George D. Loizou, James I. MacRae, Bennard van Ravenzwaay, Philippe Rocca-Serra, Reza M. Salek, Tilmann Walk, Ralf J. M. Weber

**Affiliations:** 1 School of Biosciences and Phenome Centre Birmingham, University of Birmingham, Edgbaston, Birmingham, B15 2TT UK; 2 Imperial College London, London, SW7 2AZ UK; 3 US FDA, NCTR, Jefferson, AR 72079 USA; 4 US EPA, Athens, GA 30605 USA; 5 BASF SE, 67063 Ludwigshafen, Germany; 6 Vrije Universiteit Amsterdam, Amsterdam, Netherlands; 7 Health and Safety Executive, Buxton, UK; 8 The Francis Crick Institute, London, NW1 1AT UK; 9 Oxford e-Research Centre, Department of Engineering Science, University of Oxford, Oxford, OX1 3QG UK; 10 International Agency for Research on Cancer, Lyon, France; 11 BASF Metabolome Solutions, 10589 Berlin, Germany

**Keywords:** Metabolomics, Mass spectrometry, NMR spectroscopy, Toxicology

## Abstract

Metabolomics is a widely used technology in academic research, yet its application to regulatory science has been limited. The most commonly cited barrier to its translation is lack of performance and reporting standards. The MEtabolomics standaRds Initiative in Toxicology (MERIT) project brings together international experts from multiple sectors to address this need. Here, we identify the most relevant applications for metabolomics in regulatory toxicology and develop best practice guidelines, performance and reporting standards for acquiring and analysing untargeted metabolomics and targeted metabolite data. We recommend that these guidelines are evaluated and implemented for several regulatory use cases.

## Abstract (web summary)

Lack of best practice guidelines currently limits the application of metabolomics in the regulatory sciences. Here, the MEtabolomics standaRds Initiative in Toxicology (MERIT) proposes methods and reporting standards for several important applications of metabolomics in regulatory toxicology.

## Introduction

During the last two decades metabolomics (also referred to as metabonomics, metabolic phenotyping and metabolic profiling) has become a mature technology in academic research, particularly within the biomedical and plant sciences, resulting in many thousands of research publications. While this approach has increased our understanding of the mode(s) and mechanism(s) of action of toxicity in human and environmental health^1^, its application to regulatory toxicology has been limited to date^2,3^. Although many scientists believe that this underutilisation is due to a lack of interest by the regulators working in chemical safety, this is in fact not the case. Indeed, considerable interest has been shown by regulatory scientists from the industrial chemical and food safety sectors^4–6^ who acknowledge the primary strengths of metabolomics: first, as a tool to help discover toxicological pathways and molecular key events (KEs), which could then be applied to a range of regulatory needs in predictive toxicology such as chemical grouping and read-across^7^, including the potential to discover these effects in early life-stage (e.g. fish embryos) as a non-animal alternative testing strategy; and, secondly, to provide a direct and functional measure of a cell’s or organism’s phenotype at the molecular level, which could be causally associated with an adverse outcome in the adverse outcome pathway (AOP) framework^8^. A third application for metabolomics in regulatory toxicology is its relatively unexplored use in toxicokinetics, to measure the levels of an exposure chemical and to discover any metabolic biotransformation products^9,10^.

Given these strengths—the first of which is shared by the complementary approaches of transcriptomics and proteomics, while the latter two are unique to metabolomics—why hasn’t metabolomics advanced into regulatory toxicology? Some of the reasons include a sparsity of robust case studies, a relative lack of training opportunities, and limited accessibility to the analytical and computational tools required. However, the most commonly cited roadblock to the translation of metabolomics into regulatory toxicology is the lack of best practice guidelines, including both performance standards and minimal reporting standards. For example, at the European Chemical Agency’s New Approach Methodologies (NAM) workshop in 2016, it was concluded that “There are a number of R&D needs, including a database to support metabolomics, standardisation, validation and reporting formats”^4^. While not anticipated to revolutionise the uptake of this technology into the regulatory sciences, the development of best practice guidelines that build on earlier standardisation efforts^11^ is expected to accelerate the use of metabolomics to improve the safety assessment of chemicals.

## AIM

This publication aims to describe the most relevant best practice guidelines, method performance standards, and minimal reporting standards for the acquisition, processing and statistical analysis of metabolomics data within the context of regulatory toxicology. This document has been developed as part of the MEtabolomics standaRds Initiative in Toxicology (MERIT) project. Specifically, we aim to provide regulators and other stakeholders with the first practical guidelines for interpreting the quality of metabolomics data. To facilitate this, we have considered the most immediate potential applications for metabolomics in regulatory toxicology and used these as a foundation for the development of the standards. We recognise that technologies evolve, as do regulatory needs, hence we regard this publication as a living document that will require version-controlled refinements in the future. Furthermore, the document provides a basis for the development of an international OECD guidance document and reporting template: the OECD Metabolomics Reporting Framework (MRF), initiated in 2018.

## Related standards initiatives

The guidance presented here draws considerably upon a range of best practice and minimal reporting standards that the metabolomics community has been developing for over a decade. Those significant contributions are acknowledged here, primarily the efforts in 2007 by the international Metabolomics Standards Initiative (MSI) that published ten papers introducing and describing the MSI and reporting requirements for a wide range of biological, biomedical and environmental studies, including for chemical analysis, data analysis and ontologies^11–20^. More recently, the need to continuously update these standards and for the metabolomics community to more rigorously adopt the guidelines has been highlighted^21^. The need for quality assurance in the application of metabolomics to toxicology was the topic of a workshop jointly organised by the Johns Hopkins Center for Alternatives to Animal Testing (CAAT) and the NIH Human Toxome project in 2013^22^. Regarding current activities, the MERIT project is coordinating with the recently formed metabolomics Quality Assurance and quality Control Consortium (mQACC https://epi.grants.cancer.gov/Consortia/mQACC^23^) in the writing of this document; mQACC was recently formed, following an NIH sponsored meeting in 2017 to address QA/QC issues in metabolomics. While that group’s focus is predominantly biomedical, the underlying requirements for QA/QC are similar to those required in regulatory toxicology. Through the significant momentum achieved through standards development in the related fields of transcriptomics^24–27^, RNA sequencing^28^ and proteomics (e.g. Proteomics Standards Initiative^29^), as well as by the MERIT project, the OECD Extended Advisory Group on Molecular Screening and Toxicogenomics (EAGMST) has recently launched a project to develop Omics Reporting Frameworks specifically for the purposes of regulatory toxicology. The metabolomics guidelines in MERIT are being developed in coordination with the OECD expert groups developing both the OECD Transcriptomics Reporting Framework^30^ and Metabolomics Reporting Framework.

## SCOPE

The MERIT project and this guideline publication focus on regulatory toxicology, hence on the application of the most mature, stable and proven metabolomics technologies only. Research studies in metabolomics, typically in academia, utilise a much broader array of analytical technologies and bioinformatic approaches to answer diverse questions; we are not explicitly proposing these guidelines for metabolomics research projects (although many aspects of the guidelines are in fact applicable and their application would surely benefit the quality of metabolomics research). While future applications of metabolomics can be envisioned across a wide range of regulatory practice, here we outline metabolomics case studies in substance safety for industrial chemicals and biocides, with relevant legislation including the European Union’s Registration, Evaluation, Authorisation & restriction of CHemicals (REACH) and Biocidal Products Regulation (BPR), and the United States’ Toxic Substances Control Act (TSCA). Parts of the MERIT best practice, performance standards and reporting standards will also be applicable to drug toxicology, which is currently being discussed by mQACC. As a further consideration of the scope of MERIT, we have focused on the development of the guidelines and not yet on their actual implementation; i.e., we have not created and implemented a database schema for the minimal reporting standards. This is an important future task and one that can be addressed by developing an OECD Harmonised Reporting Template (OHT; https://www.oecd.org/ehs/templates) for metabolomics, building on the new OECD Metabolomics Reporting Framework project.

**Fig. 1:**
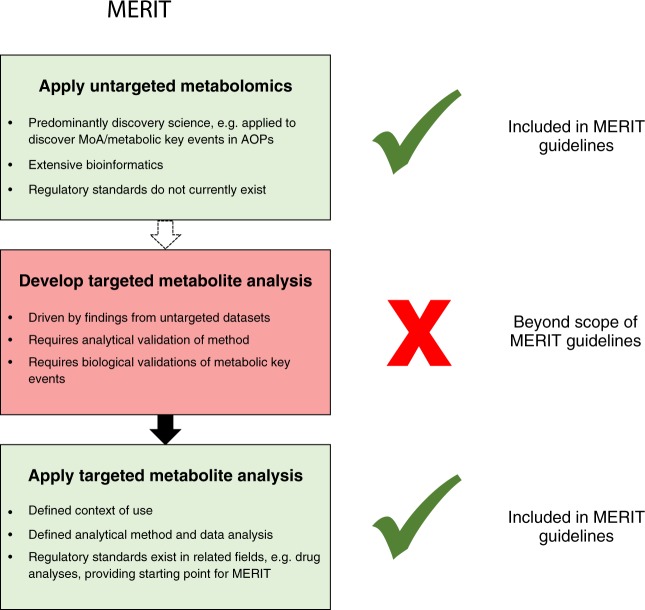
Types of metabolite analyses in regulatory toxicology and scope of the MERIT project. Dotted arrow indicates potential translation of knowledge from untargeted to targeted assays, although targeted assays can be developed and applied without an initial metabolomics study

Considering the scope of this guideline, we have focused specifically on the applications of untargeted metabolomics and targeted metabolite analysis in regulatory toxicology, not on the development of methods (Fig. 1 and Table 1). While untargeted metabolomics aims to achieve broad metabolite profiling of a given sample, targeted metabolomics aims to quantify a small number of pre-selected metabolites, e.g. the study of fatty acid biosynthesis. We also include the application of semi-targeted metabolomics to regulatory toxicology, a hybrid approach that combines untargeted and targeted analyses (Table 1). As recognised within the metabolomics research community, untargeted and targeted approaches can be strongly related, with discovery driven (or hypothesis generating) untargeted metabolomics naturally translating into targeted (or hypothesis testing) metabolite assays for downstream utilisation. However, this translation requires extensive analytical development and validation as well as biological validation of the targeted biomarkers, which is outside of the scope of the MERIT project (Fig. 1).

**Table 1:** Definitions of key terms used in the best practice guidelines and reporting standards

Term	MERIT definition
Metabolomics	Systematic study of endogenous metabolites and the biochemical processes that they are involved in, within a cell, tissue, or organism.
Endogenous metabolite	Precursor, intermediate, or a product of metabolic biochemical reaction, produced by the host cell or organism.
Untargeted toxicokinetics	Measurement of the absorption, distribution, metabolic biotransformation, and/or excretion of a chemical specifically as part of an untargeted metabolomics toxicity study.
Untargeted	Analytical assay in which the analytes are not predefined and are typically unidentified during initial data acquisition. The approach attempts to measure the broadest range of endogenous metabolites possible. The assay is semi-quantitative.
Targeted	Analytical assay in which the analytes are predefined and identified to MSI level 1 (see supplementary note 11). The assay can be quantitative or semi-quantitative.
Semi-targeted	An assay which combines both targeted and untargeted approaches. Some analytes will comprise a targeted panel (identified to MSI level 1), but the remaining data is acquired and treated as untargeted. The approach attempts to measure the broadest range of endogenous metabolites possible. The assay is at least semi-quantitative.
Semi-quantitative	An assay in which only relative amounts of each analyte can be compared—e.g. a given analyte may be twice the concentration in one sample than another, though the absolute concentrations are not known.
Quantitative	An assay resulting in absolute concentration information for each analyte (e.g. in M or μg/L).
Quality assurance	A set of procedures that are done in advance of analysis and that are used to improve the quality of data.
Quality control (QC)	A set of activities that a laboratory does during or immediately after analysis that are meant to demonstrate the quality of project data.
System suitability QC	QC sample type used to demonstrate the analytical system is fit for purpose and working within specification.
Intrastudy QC (previously termed pooled QC)	QC sample type used within one study with multiple purposes, primarily to assess (and potentially correct for) intrastudy reproducibility.
Intralab QC	QC sample type used within one laboratory to assess (and potentially correct for) any differences between separate studies.
Interlab QC	QC sample type used across multiple laboratories to assess (and potentially correct for) any differences between laboratories.
Process blank	QC sample type used to measure any interfering signals (i.e. contaminants) that may arise from the ‘process’, e.g. extraction, such that these contaminant signals can be removed from a dataset.

## Participants

The MERIT project brought together a team of international experts from industry, government agencies, regulators and academia from across Europe and the United States, including the US Environmental Protection Agency, US Food and Drug Administration, UK Health and Safety Executive, BASF, Imperial College London, University of Birmingham, VU University Amsterdam, The Francis Crick Institute, and the Metabolomics Society Data Standards Task Group. Following the development of a draft guidance document by the core team, it was opened to international consultation (again including a wide range of stakeholders).

## Scenarios for regulatory application of metabolomics

**Fig. 2:**
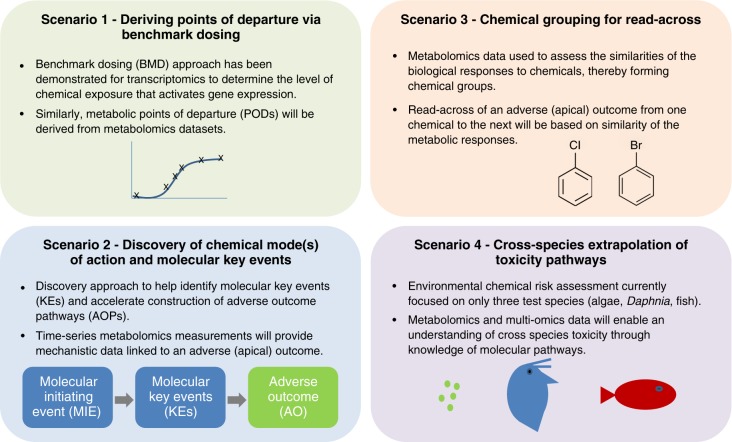
Four scenarios for the application of metabolomics to regulatory toxicology

As introduced above, while many scientists and regulators have recognised the considerable potential value of metabolomics in regulatory toxicology, there are still relatively few (publicly available) examples of metabolomics addressing specific regulatory questions. Over the last decade, industry has taken a lead in demonstrating the value of metabolomics^31–35^, particularly to extend the concept of chemical grouping from one based on the similarity of chemical structures to being based on the similarity of metabolic signatures from mass spectrometry-based metabolomics studies. While these advances are promising, as was highlighted at the European Chemical Agency NAM workshop in 2016^4^, the sparsity of detailed metabolomics case studies in regulatory toxicology led the MERIT project to two conclusions: first, that this publication should review the most immediate applications for metabolomics in regulatory toxicology, and second that it should define best practice guidelines and performance standards that can cover a range of immediate and future case studies. Below we introduce four of the most likely regulatory scenarios for metabolomics in toxicology, providing an anchor for the standards development (Fig. 2).

### Scenario 1: Deriving points of departure via benchmark dosing

To ensure public safety and environmental quality, regulatory agencies are required by law to undertake safety assessments of potential hazards^36^. The identification of a reference point (RP), also known as a point of departure (POD), is common to all risk assessments of chemicals with threshold effects. A RP/POD is defined as the point on a toxicological dose–response curve established from experimental data that corresponds to an estimated no effect or low effect level, which ultimately is used to derive a toxicological reference dose. The benchmark dose (BMD) approach fits a dose–response model(s) to the complete dose–response dataset to derive a RP/POD for risk assessment^37–39^. This response could be adverse or not, depending on its association with an apical endpoint. Although not yet applied to metabolomics data, BMD has successfully been applied to rodent transcriptomics data allowing a comprehensive survey of the transcriptional changes associated with chemical exposure as well as estimating reference doses at which some cellular processes are altered^40,41^. The derivation of a POD by applying the BMD approach to metabolomics dose–response data has the potential to be adopted in chemical risk assessment, because of the generic utility of RP/PODs in current regulatory practice^5^ and the close association of metabolic phenotypes with regulatory apical endpoints^42,43^. This latter point is particularly noteworthy and offers a benefit to metabolomics that is not shared, for example by transcriptomics, although care would be needed to interpret a metabolic POD as adverse or not. Measurement of a metabolic POD could also be used for prioritisation, i.e., to determine the chemical(s) that induces a statistically significant metabolic effect at the lowest exposure concentration, and then prioritise that chemical for more extensive testing.

Best practice and performance standards for a metabolomics BMD investigation would need to cover all aspects of the workflow, from experimental design, QA/QC procedures, sampling, metabolite extraction, measurement of metabolites, data processing, post-processing and statistical analysis. This scenario requires the use of both a sufficiently high number of dose groups and an adequate sample size per dose group, in order to model the response of each metabolite as a function of external dose. Typically, 6–8 doses over a relatively wide range are necessary, from just below the no observed effect level of an apical endpoint (due to the higher sensitivity of the metabolomics measurements^44^) to a maximum tolerable dose or cytotoxic dose, dependent upon the test system. One of the important steps in this type of analysis is to attempt to identify the subset of metabolites that demonstrate dose–response behaviour and that ultimately may be used to estimate the benchmark dose at which the abundance of the metabolite significantly deviates from normal levels. While identified metabolites could provide a more robust biomarker, applying the BMD approach to unannotated peaks in a mass spectrum or NMR spectrum could in principle meet regulatory standards if the peak can be consistently measured across laboratories. Reporting the level of confidence in metabolite annotation or identification is critical. Greater confidence in the derived POD value would be obtained if several metabolites within the same metabolic pathway showed a similar response, equivalent to gene expression data being mapped to the same gene ontology category. Case studies demonstrating the application of metabolomics to derive PODs using the BMD approach are eagerly awaited.

### Scenario 2: Discovery of mode(s) of action and molecular key events

Adverse outcome pathways (AOP) were first developed by the US EPA in 2010 as a knowledge management tool for describing pathways associated with toxicity and have since gained widespread international momentum, including as part of the OECD’s chemical safety programme^45,46^. An AOP is a knowledge framework that represents the causal relationships between the first interaction of a chemical with a biological system, known as the molecular initiating event (MIE), and a series of higher order events (at the molecular, cellular, tissue levels, etc.—termed key events; KEs), which ultimately lead to an adverse outcome (AO) at the level of an individual or population (where adverse outcome is synonymous with a regulatory apical endpoint)^47–49^. While an increasing number of draft AOPs are under construction, only six have been fully endorsed to date by the OECD’s Task Force for Hazard Assessment and Working Group of National Coordinators of the Test Guidelines programme. Since omics technologies are designed to measure the responses of tens of thousands of genes and many of their downstream products (i.e. metabolites) in a hypothesis free manner, they are ideally positioned to accelerate the discovery of molecular toxicity pathways (and molecular KEs, which can also be referred to as biomarkers) and therefore to accelerate the construction of AOPs^50^.

While the application of metabolomics to molecular KE discovery within a regulatory scenario requires consideration of QA and QC standards along with data management for the whole workflow, from experimental design through to statistical analysis, it is again the experimental design that requires particularly careful consideration. For this scenario we propose that both time-series and dose–response measurements are essential, for discovering chemical mode(s) of action^51^. First, the response of a cell to chemical exposure is a time dependent process, and so to characterise the series of KEs, it is necessary to measure the system at several time points, capturing the evolution of the molecular changes, i.e. the toxicodynamics^52^. Second, to understand the essentiality of a series of molecular KEs, knowledge of the sequence of those molecular changes is necessary. Such an approach can also help to infer causality between KEs, including whether a particular molecular KE is predictive of an adverse outcome, providing a deeper understanding of the mechanism. Third, classification analysis of metabolic responses (to group chemicals) that incorporates time-series data should in principle have greater predictive capability than classification models that ignore time, as it has been reported for classifying disease outcomes in biomedical research^53^; this is particularly relevant to the following scenario. A further important point about molecular KEs is that these may be more informative (and less variable) when defined at the molecular pathway level, and not as individual genes or metabolites. The former representation should be less susceptible to the variability in individual measurements and would also provide an opportunity to integrate gene expression and metabolism within a pathway. Currently this remains an active research topic. What is clear is that only through appropriate reporting of metabolomics studies for biomarker and molecular KE discovery will AOP reviewers and regulators be able to assess the reliability of the conclusions.

While scenario 2 focuses on the mode(s) of action (or toxicodynamics) of a chemical, the experimental design proposed here—i.e. to measure the biological system at several time points to capture the evolution of the molecular changes—can in principle reveal equally interesting insights into the toxicokinetics, that is, the absorption, distribution, metabolism and/or excretion of the exposure chemical. Indeed, relationships between the toxicokinetics and toxicodynamics of a chemical could be extracted from the same untargeted metabolomics dataset. Metabolite prediction software such as BioTransformer^54^ or SyGMa^55^ can aid in the identification of biotransformation products of chemicals or drugs. Here we refer to the study of a parent chemical and/or its metabolic biotransformation products within a metabolomics experiment as untargeted toxicokinetics (Table 1).

### Scenario 3: Chemical grouping for read-across

Traditional read-across approaches in regulatory toxicology utilise the similarity of molecular structure between two or more chemicals to infer the endpoint toxicity of target substance(s) based on the existing knowledge of the endpoint toxicity of an analogous (or source) chemical^56^. While this approach is currently the most often used alternative to animal testing within Europe, it often leads to rejection of the risk assessment dossiers by the European Chemicals Agency (ECHA)^57^. Consequently, it is recognised that the quality of read-across needs to improve, i.e., more scientific evidence is required to support the read-across argument^58–60^. BASF have demonstrated a promising extension of this read-across approach in which they utilised rodent metabolomics data to assess the similarities of the responses of chemicals, and read-across the endpoint toxicity based on the similarity of the metabolic effects^3^. As mentioned above, the introduction of a time course experimental design may further enhance our ability to assess the similarities of the responses to a series of chemicals, thereby grouping those chemicals more accurately according to their molecular responses (and KEs).

For this scenario, several publications and workshop reports provide a basis for developing best practice and associated performance and reporting standards^31,57,61^. For example, scientists applying metabolomics to read-across concluded that significant efforts to extensively investigate and then attempt to control metabolic variability, whether that be of biological or technical origin, is essential^3^. Furthermore, they highlighted the importance of reference substances that serve as toxicological positive controls, i.e. chemicals that can be relied upon to induce a strong, consistent metabolic effect. Such control experiments should be regularly performed, and the variability of the responses can serve as an indicator of reproducibility of the entire workflow, from exposure to metabolomics measurements and data analysis. One further recommendation is that procedures should be documented in standard operating procedures and followed meticulously^62,63^, with any exceptional deviation from an SOP recorded and justified. The best practice, performance standards and reporting standards described below attempt to meet these recommendations. Even for this grouping and read-across scenario, for which a case study with phenoxy herbicides has been published^3^, there is further scope for developing the metabolomics workflow and potentially improving the best practice. For example, alternative strategies could be evaluated to derive quantitative measures of similarity between the metabolic responses to a source chemical and set of target chemicals in the read-across study (i.e. whole metabolic signatures or metabolic pathways, through to a series of metabolic KEs or biomarkers). A further development would be the use of untargeted metabolomics to inform on basic toxicokinetic parameters (Table 1), for example which metabolic biotransformation products are formed (and their relative toxicities versus the parent substance) and potentially measuring the clearance rate of the parent substance, to further support a read-across case^56^.

### Scenario 4: Cross-species extrapolation of toxicity pathways

While all the above scenarios are applicable to environmental risk assessment, most omics studies on BMD, AOPs and KE discovery, and read-across have been focused more towards human chemical safety using rodent and in vitro models. Within Europe, chemicals that are manufactured or imported in sufficiently high volume must also undergo environmental risk assessment, yet this is done using a very simplified model of the environment. The toxicity response of a selected number of species (typically three, spanning three trophic levels in the aqueous environment) is used to assess the hazard of individual substances. This typically includes algal toxicity tests (primary producers), *Daphnia* tests (primary consumers) and fish tests (secondary consumers). One of the most important questions in environmental risk assessment is to what extent are toxicity assessments in one species applicable to other species?^64,65^ Applying omics technologies to study how genes, proteins and metabolites interact and respond to chemical exposures in a selected group of model organisms could reveal the phylogenetic origins of the toxicological pathways that are predictive of adverse outcomes. Such comparative approaches are already showing promise for characterising interspecies differences in MoAs, which intriguingly is applicable to both environmental and human health risk assessment^66–68^. However, translating these exciting developments towards regulatory applications in the risk assessment of chemicals requires that the omics investigations are conducted rigorously. Again, for metabolomics, all aspects of the workflow from experimental design, QA/QC, sampling, metabolite extraction and measurement, data processing and statistical analysis, should all meet the performance standards and reporting standards described here.

## Best practice, performance and reporting standards

**Fig. 3:**
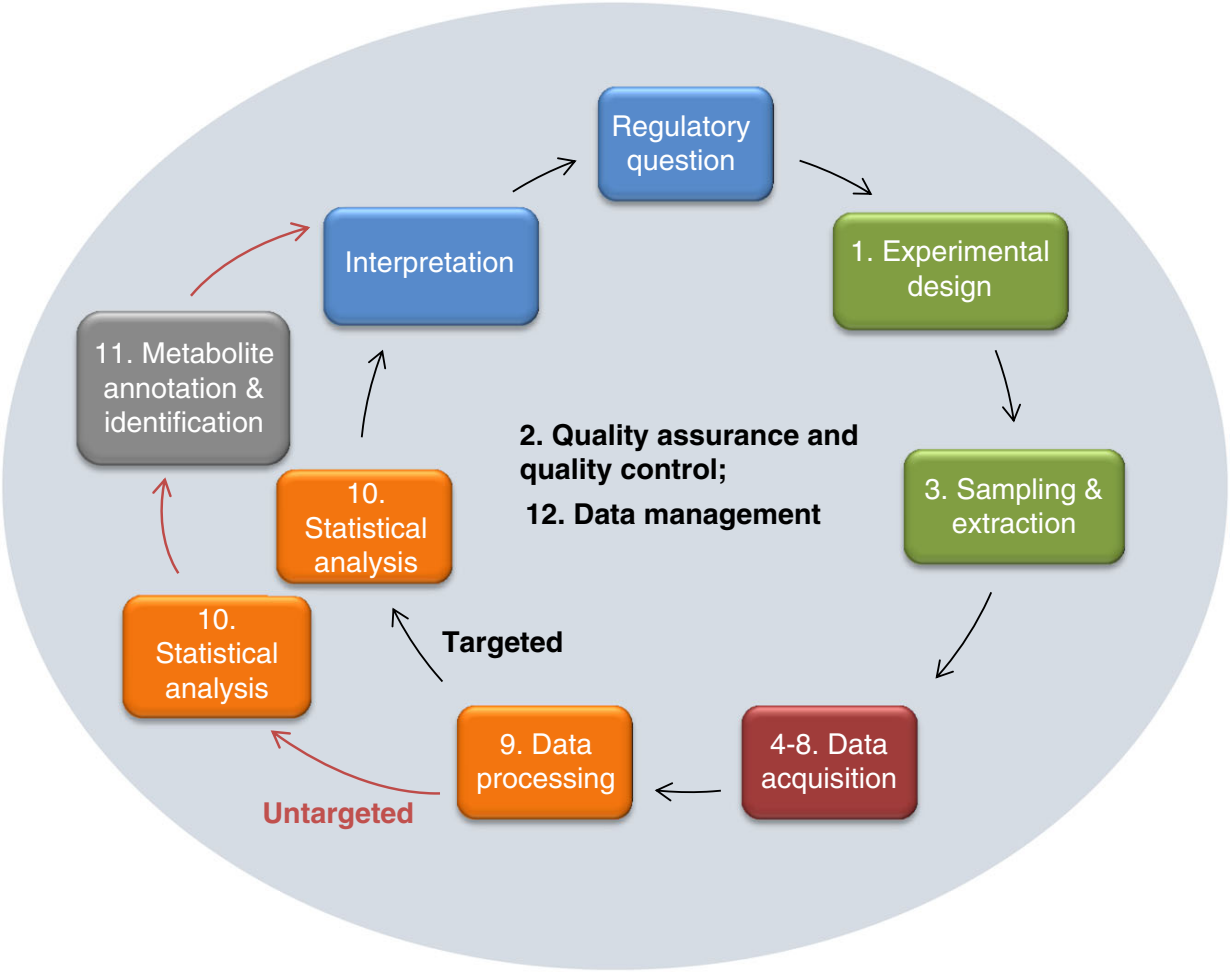
Overall workflows for untargeted metabolomics and targeted metabolite analyses. A further approach called semi-targeted analysis represents a hybrid of targeted and untargeted assays. Numbers refer to the Supplementary Notes that describe each section of the workflows

**Fig. 4:**
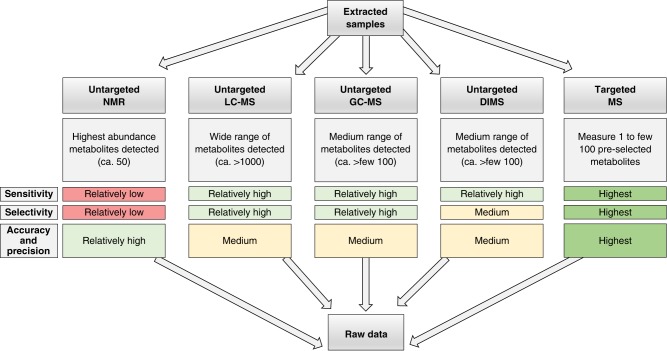
Differing capabilities of the analytical methods used in metabolomics. Semi-targeted methods combine both targeted and untargeted approaches. Direct infusion mass spectrometry (DIMS) can be targeted or untargeted

The best practice, performance and reporting standards have been developed in 12 sections that consider all aspects of a metabolomics study in regulatory toxicology, summarised in Fig. 3, starting with the regulatory question or use case. The first section (Supplementary Note 1) describes those aspects of the experimental design of a regulatory toxicology study that are most critical for metabolomics. This includes detailing the test species, the importance of defining what is metabolically normal for the untreated test species, the chemical exposures and the use of positive biological controls, sample size, the design and number of sampling times, and randomisation and batching of samples. Supplementary Note 2 defines quality assurance and quality control requirements with an introduction to five types of QC samples (system suitability QC, intrastudy QC, intralab QC, interlab QC and process blank). The purposes, performance standards, compositions and sources of these QC samples are described, along with their practical utilisation. Next, in Supplementary Note 3, the sampling and metabolite extraction for the sample types most likely to be encountered in a regulatory toxicology study are presented, including washing, quenching, metabolic extraction, and sample reconstitution in solvent for analysis. Supplementary Notes 4–7 describe technique-specific sample preparation, QC usage, data acquisition and processing for the four most widely applied analytical methods in untargeted metabolomics, comprising NMR spectroscopy, LC-MS, GC-MS and DIMS, respectively. The widespread use of these four methods was documented via international surveys^69,70^. Figure 4 highlights the differing capabilities of the analytical methods which are sometimes applied in parallel to the same study samples. Data acquisition and processing for targeted mass spectrometry assays, including reference standards for identification and quantification, and LC-MS/MS and GC-MS/MS, is presented in Supplementary Note 8. This topic has been included due to the anticipated future importance of targeted metabolite analysis in regulatory toxicology.

**Fig. 5:**
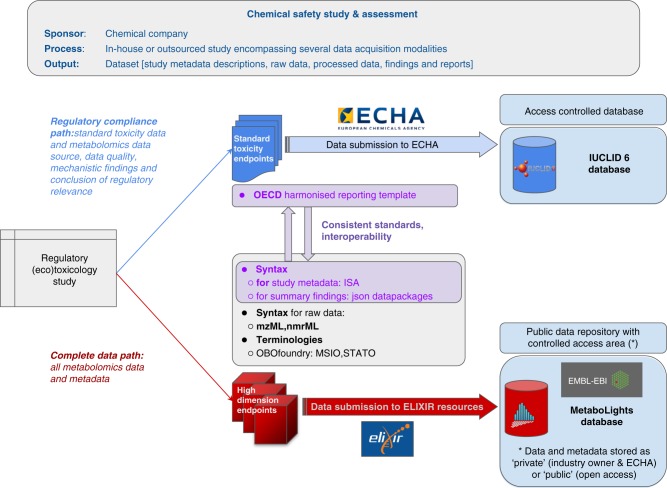
Proposed management strategy for metabolomics data from a regulatory toxicology study. The strategy benefits from several existing access-controlled and public resources and would allow compliance standards to be checked by the chemical regulator (illustrated here for Europe) as well as open the potential for (metabol)omics data reuse subject to approval by the industry owner. One area of development is to align metadata standards between the regulatory compliance and complete data paths

The next steps in the untargeted metabolomics and targeted metabolite analysis workflow are largely instrument independent, including data post-processing (Supplementary Note 9) and statistical analysis (Supplementary Note 10). Best practice and reporting standards for data post-processing cover the topics of signal intensity drift correction and batch correction, imputing missing values, normalisation, and strategies for filtering variables (i.e. *m*/*z* features) to ensure high quality datasets. The particularly important topics of intrastudy QC analysis and biological sample outlier detection and removal are described in Supplementary Note 10, followed by scaling/transformations and the application of uni- and multivariate statistical analyses, the latter covering validation methods and feature selection. To facilitate interpretation of the statistical results in terms of the regulatory question (Fig. 3), this section also introduces the statistical analyses specific to the regulatory questions summarised in Fig. 2. Supplementary Note 11 describes the current minimal reporting standards for levels of confidence in metabolite annotation and identification, which are critical for robustly appraising the validity of any biochemical interpretation within a metabolomics study. Finally, since no best practice yet exists for the management of metadata and experimental data from metabolomics studies within regulatory toxicology, in Supplementary Note 12 we propose a new strategy and architecture for integrated data management, sharing and exploitation. This framework, summarised in Fig. 5, includes controlled vocabularies, file formats and databases for metabolomics raw data and metadata.

## Conclusions and future directions

From an academic scientist’s perspective the field of metabolomics (under its various synonyms) is most definitely mature, and some would argue in excess of 20 years old (though continuing to develop in tools, applications and impact). From a regulator’s perspective it has only just become a NAM—a New Approach Methodology^4^. The time has now come to close this gap and to evaluate the potential benefits of metabolomics specifically in the context of regulatory toxicology. The first steps on that journey have begun, and the relevant communities are now talking regularly. It is our intention that this guidance document prompts further discussions in these and related communities, and actions are taken to address the remaining challenges. Arguably the most urgent challenge is to develop exemplar case studies for the application, interpretation and reporting of metabolomics data as part of larger toxicity studies, co-developed by regulatory scientists, and including other NAMs alongside traditional assays and measurements. We are not proposing that metabolomics data should routinely stand alone, but instead contribute to a weight of evidence approach, i.e. as a component of Integrated Approaches to Testing and Assessment (IATA)^71,72^. In this document we have proposed four such case studies or scenarios that we recommend are worked up as examples for demonstrating the value of metabolomics. Furthermore we have identified the need for, and recommend the development of, more detailed guidelines for the interpretation of the metabolomics data from each of these case studies, thereby providing more substance to the blue Interpretation box in Fig. 3.

What is clear is that the metabolomics community knows how to conduct and report high-quality scientific studies—using both untargeted metabolomics and targeted metabolite analysis. Much of the content of this guidance document reflects that knowledge. For example, see Supplementary Table 4, which summarises some of the minimum criteria for best practice and reporting for the application of these two related approaches. What requires further development is precisely how this best practice is implemented into specific regulatory scenarios, such as those we introduced in this paper, including MoA/KE discovery, deriving points of departure for helping to derive health-based guidance values, etc. We strongly encourage the academic, industry and regulatory communities to work together to develop exemplar case studies for these specific regulatory applications. This should include how regulatory decision-making could be achieved based on the types of statistical analyses presented in supplementary note 10, in a transparent, simple yet robust manner.

A further challenge that these communities need to address is determining optimal strategies for managing ‘omics data and metadata derived from regulatory toxicology studies. Some would argue that the benefits of open data formats, that are now widely accepted and adopted in research communities, should be applied in regulatory toxicology. Ensuring that data are FAIR (findable, accessible, interoperable and reusable^73^) would increase the transparency of chemical safety regulation. Yet REACH places the responsibility of ensuring substance safety on those synthesizing and using chemicals in Europe, hence the metabolomics data would be owned by industry who may be unwilling to share such data publicly. Perhaps industrial innovators will want to make their metabolomics data FAIR? Perhaps future innovative legislation will require that ‘omics data derived from chemical safety testing must be made public.

We believe the most productive route forward is to create exemplar projects and case studies that seek to unite the expertise, needs and solutions across the metabolomics research, industrial and regulatory toxicology communities. Indeed, the ECETOC-supported MERIT project, although focused on the production of a set of best practice guidelines, has additionally provided a forum for scientists from these communities to unite around a common purpose. Networks have expanded, much has been learnt by all parties, and tangible best practice, performance and reporting standards have been proposed. We look forward to engaging with the wider community to work towards acceptance of these guidelines, developing them further as part of the new OECD Metabolomics Reporting Framework project, and ultimately to a time when metabolomics data are accepted routinely as part of regulatory toxicology studies.

## Supplementary information

Supplementary Information
