# Supplementary material for: Use cases, best practice and reporting standards for metabolomics in regulatory toxicology
Source: Nat Commun. 2019 Jul 10;10:3041. doi: 10.1038/s41467-019-10900-y (PMC6620295; doi:10.1038/s41467-019-10900-y)
Supplement: Supplementary file 1 — Supplementary Information [file 41467_2019_10900_MOESM1_ESM.docx]

# Supplementary Information

# Use Cases, Best Practice and Reporting Standards for Metabolomics in Regulatory Toxicology

Mark R. Viant^1,✝,^, Timothy M. D. Ebbels^2,✝^, Richard D. Beger^3^, Drew R. Ekman^4^, David J. T. Epps^1^, Hennicke Kamp^5^, Pim E. G. Leonards^6^, George D. Loizou^7^, James I. MacRae^8^, Bennard van Ravenzwaay^5^, Philippe Rocca-Serra^9^, Reza M. Salek^10^, Tilmann Walk^11^, Ralf J. M. Weber^1^

*^1^School of Biosciences and Phenome Centre Birmingham, University of Birmingham, ^2^Imperial College London, ^3^US FDA, ^4^US EPA, ^5^BASF SE, ^6^Vrije Universiteit Amsterdam, ^7^Health and Safety Executive, ^8^The Francis Crick Institute, ^9^Oxford e-Research Centre, University of Oxford, ^10^International Agency for Research on Cancer, ^11^BASF Metabolome Solutions*

^✝^These authors contributed equally: Mark R. Viant, Timothy M. D. Ebbels

Correspondence and requests for materials should be addressed to: M.R.V. (email: m.viant@bham.ac.uk)

| **Supplementary Note** | **Title** | **Pages** |
| --- | --- | --- |
| 1 | Experimental designs | 3 – 10 |
| 2 | Analytical quality assurance and quality control | 11 – 17 |
| 3 | Sampling and metabolite extraction | 18 – 23 |
| 4 | Data acquisition and processing: Untargeted NMR | 24 – 28 |
| 5 | Data acquisition and processing: Untargeted LC-MS | 29 – 32 |
| 6 | Data acquisition and processing: Untargeted GC-MS | 33 – 36 |
| 7 | Data acquisition and processing: Untargeted DIMS | 37 – 39 |
| 8 | Data acquisition and processing: Targeted MS assays | 40 – 43 |
| 9 | Data post-processing | 44 – 49 |
| 10 | Statistical analysis | 50 – 56 |
| 11 | Metabolite identification | 57 – 58 |
| 12 | Management of data and metadata from regulatory metabolomics studies | 59 – 68 |

## Supplementary Note 1. Experimental designs

Many factors must be considered to ensure that a robust experimental design is employed for regulatory toxicology studies. Here we focus primarily on those factors that are specific and/or highly relevant to metabolomics. These include detailing the test species, the importance of defining ‘what is normal’ for the metabolome of the untreated test species, chemical exposures and the use of positive biological controls, the design and number of sampling times, sample size and statistical power, and randomisation and batching of samples. We refer the reader to other documents for guidance on generic toxicity testing, e.g. the OECD Guidelines for the Testing of Chemicals (<http://www.oecd.org/chemicalsafety/testing/oecdguidelinesforthetestingofchemicals.htm>). We cannot yet define the optimal experimental designs for the four scenarios introduced above for regulatory application of metabolomics due to a relative lack of such studies reported in the literature. Based on research currently being undertaken, it is anticipated that optimal designs will be developed in the next two or three years, building on the more generic factors described below.

### Test species and defining ‘what is normal’ metabolism

While a wide range of animal and plant species and *in vitro* test systems are utilised in metabolomics studies in academic research, far fewer test systems are used in regulatory toxicology. For example, for regulatory assessments of relevance to human health, *in vivo* studies remain focused on rodents (e.g. commercially bred rat strains, such as Sprague Dawley, Wistar, or F344), although momentum is building to transition towards *in vitro* human cell line models (e.g. HepG2, HepaRG ^1,2^). For environmental risk assessments, the test systems often include various fish species, waterflea (*Daphnia* spp.) and algae (e.g. *Chlamydomonas* spp.). In principle, the biochemistry of any species can be investigated using metabolomics. However, best practice is for studies to either focus on already well (metabolically) characterised test systems, or that the test system to be used is thoroughly characterised, which is in itself a significant undertaking. The advantage of the former approach is that the metabolomics data will better align with data generated by traditional regulatory tests. Indeed, studies are now being reported that include both metabolomics and traditional apical endpoints ^3,4^.

A particular challenge in metabolomics studies stems from the susceptibility of the cellular or organismal metabolome to respond to the environment. On one hand, this responsiveness of the metabolome provides value for detecting the biochemical changes induced by toxicant exposure, yet it can also lead to unwanted perturbations to metabolite levels caused by a wide range of confounding factors. The following parameters will affect metabolism and should be considered in the selection of the test species and its husbandry or culturing: genetic background ^5,6,7^, age ^8^, life stage ^9^, diurnal cycle ^10^ and sex ^11^. In addition, the test species’ local environment can affect metabolism, the most obvious factor being diet. It is therefore critical that procedures within a laboratory are standardised and reported.

Draft minimum reporting requirements for biological samples and procedures within a mammalian metabolomics study ^12^ and for *in vitro* studies ^13^ were reported in 2007 by the MSI. The mammalian study requirements comprised of four parts: subject description, husbandry, experimental design and sample collection (the latter two are discussed in subsequent sections). Further reporting requirements have been recommended in the ToxRTool ^14^.

Test species description:

**REPORT**: Model description (species/strain for *in vivo*; species/cell type(s) for *in vitro*); supplier or source of test system(s); age range and/or developmental stage (*in vivo*); sex (*in vivo*); weight range (*in vivo*).

Husbandry/culturing:

**REPORT**: Housing; light cycle; temperature; feeding regimen; acclimation duration (for *in vivo*)

**REPORT**: Well-plate or flask type; temperature; CO_2_/O_2_ conditions; humidity; culture media including use of serum and/or antibiotics (for *in vitro*).

Defining ‘what is normal’ metabolism in the test species:

Best practice requires the use of *untreated negative control samples* (i.e. samples not exposed to a test chemical or carrier solvent) in every study. This is essential for enabling the metabolism of the untreated controls to be compared across independent experiments, over a period of many years. While the observation that control samples vary between experiments does not directly implicate changes in the test species or husbandry/culturing, such observations should be followed up to attempt to discover why the measurement of the metabolism of control samples is varying. Conversely, the observation that control samples do not vary significantly between independent experiments indicates a consistency in the test species and husbandry/culturing. Depending on the regulatory question, and hence experimental design used, the baseline measurements in control samples should mirror the sampling and analyses applied to the exposed test species. For example, untreated control samples should be age-matched, of the same sex, and sampled over the same time points as the treated samples (see Section 1.3). Over multiple studies, this builds an invaluable source of data, the metabolome equivalent to the historical data derived from classical toxicology assessments, both for the laboratory undertaking the work (for interstudy comparisons) as well as for interlaboratory comparisons.

**REPORT**: Metabolomics measurements (see multiple sections below for how to report) for age-matched, sex-matched, untreated negative control samples, to define ‘what is normal’.

### Chemical exposures and defining ‘metabolic response of a positive control’

For metabolomics studies in toxicology, careful consideration must be given to the exposure chemical(s) (and its potential metabolic biotransformation products) as well as the use of anaesthetics and/or dosing vehicles. This is because any low molecular weight chemicals (i.e. <1200 Da) added to the test system might be detected in a metabolomics assay, potentially complicating the spectrum of the endogenous metabolic phenotype. While such practices may be unavoidable, for example many test chemicals require dissolution into an organic solvent (also known as carrier solvent or dosing vehicle), it is essential that any ‘external’ (exogenous) chemicals used in the exposure study are reported, including their identities and concentrations.

Utilising a metabolomics assay to detect the exposure chemical and any metabolic biotransformation products is currently a largely unrecognised advantage of this technology. Indeed, metabolomics has the potential to simultaneously provide information on toxicokinetics, including, for example, the clearance rate of the exposure chemical (dependent on the experimental design) as well as discovering the identities of (and measuring formation rates) of biotransformation products ^15^. No best practice for the application of untargeted metabolomics to toxicokinetics - which we term *untargeted toxicokinetics* - yet exists. However best practice does exist for traditional toxicokinetics using targeted analytical assays ^16^.

The range of chemical exposure concentrations used depends on the regulatory question being addressed. For example, a greater number of concentrations will be needed to derive a metabolic health-based guidance value (i.e. point of departure, such as a no observed metabolic effect level) than typically needed to discover and/or classify MoA(s) ^16,17,3^. Hence, no generic best practice can be provided for the number of concentrations investigated (see Scenarios for Regulatory Application of Metabolomics, above), though it is important to report the exposure design as well as the justification for the dosing regimen used. Due to the importance of selecting the appropriate number of biological replicates for a metabolomics study, this is addressed separately below.

Test chemical:

**REPORT**: Identity; source; concentration(s) and justification for dosing regimen used; description of stock solution (including dates of preparation and, if relevant, dilution).

Other exogenous chemicals:

**REPORT**: Identity; source; concentration(s); description of stock solution (as above); purpose of exogenous chemical.

Defining ‘metabolic response of a solvent control’:

For studies in which a carrier solvent (or dosing vehicle) is used, best practice requires the use of a *solvent/vehicle control* (i.e. a subset of the test organisms/cells that are exposed to the solvent/vehicle alone). This provides a comparator dataset for any metabolic perturbations measured in response to the test chemicals.

**REPORT**: Metabolomics measurements (see multiple sections below for how to report) for solvent/vehicle control samples.

Defining ‘metabolic response of a positive control’:

Best practice requires the use of a *positive control(s)* (i.e. a subset of the test organisms/cells that are exposed to defined concentration(s) of a reference test chemical(s)) which is included across all studies). This enables the metabolic perturbation(s) caused by that reference chemical (relative to the untreated control or solvent control samples) to be compared across independent experiments. The observation of consistent metabolic perturbations - relative to those from prior observations of the positive controls - provides a measure of the repeatability of the whole study, including the test species, exposure experiment and metabolomics assay.

**REPORT**: Metabolomics measurements (see multiple sections below for how to report) for positive control organisms that were consistently exposed to a reference test chemical(s).

### Design and number of sampling times

Multiple factors should also be considered when sampling material for metabolomics analyses, be that cells, media, biofluids, tissues or (small) whole organisms. Given the importance of the process used for sampling, and the need for immediate quenching of metabolism, the best practice for this component of a study is described below. Here we introduce contemporary ideas for designing optimal sampling strategies, which will depend on the regulatory question being addressed. Furthermore, sampling strategies are constrained by multiple operational, ethical and financial factors. For example, repeated sampling in an *in vivo* study must utilise a readily accessible (and non-lethal) sample type such as urine or plasma; also, chronic toxicity experiments must include feeding, which can complicate the interpretation of metabolic responses with animals altering their food intake as a result of chemical exposure, and in turn, indirectly affecting their metabolism.

A further consideration is that baseline metabolism in both *in vivo* and *in vitro* test systems will change over time. This has been demonstrated most extensively in metabolomics studies for early life-stage test species, including for example Japanese medaka ^9^, zebrafish ^18^ and *Drosophila ^19^*. Indeed, developmental metabolic changes can be larger than those induced by a test chemical, complicating the interpretation of metabolomics data.

While the optimal design and number of sampling times for metabolomics measurements will depend on the regulatory question being asked, it is important to use *time-matched* control samples to help deconvolve chemical-induced changes from underlying developmental processes (this is in addition to ensuring samples are age-matched and sex-matched, discussed in Section 1.1). As argued in Scenario 2 - Discovery of chemical mode(s) of action or molecular key events, above, time-series measurements should provide considerable value for discovering a chemical’s MoA. However, ethical and financial factors may limit the scale of time series studies. There is currently no consensus on the minimal number of time points required although some rudimentary arguments provide a framework. Specifically, with the objective of determining how metabolites and/or metabolic pathways change over time, in some cases three time points may provide sufficient insight into the evolution of the effect(s). However, five time points have been argued to be the minimum, with closer to ten time points allowing more sophisticated time series analyses (Ben Brown, pers. comm.). Such experiments would be most feasible in high throughput *in vitro* toxicity testing. For some other regulatory scenarios, fewer time points are required. For example, metabolomics data has been used at a single time point (28 days in rat) for MoA classification, which was made feasible by earlier studies that determined this as a suitable time point. Observed differences in endogenous metabolism (toxicodynamics) should be considered in light of potential differences in the toxicokinetics between chemicals.

Sampling time:

**REPORT**: Time(s) of sampling.

Other biological measurements at sampling (optional, study specific):

**REPORT**: Food consumption; weight of organism.

### Sample size and statistical power

Any experimental design requires consideration of sample size to avoid unnecessary waste of resources and to comply with ethical principles related to animal use or human participation. Power calculations in omics sciences, and particularly metabolomics, are more complex than those for traditional assays for several reasons: 1) due to the untargeted nature of the approach, the identity and number of metabolites responding to the treatment is unknown *a priori*, 2) the variation of the metabolic response(s) in the population is usually unknown *a priori*, 3) effect sizes are usually unknown *a priori,* and 4) many multivariate statistical tools (e.g. PLS-DA) are too complex to use traditional parametric approaches, so that numerical techniques must be used. In addition, these tools are often used in the exploratory phase of analysis, where expected effects and their sizes are not known. Thus, although power calculations are important, one should avoid over-reliance on their output.

Some of the above problems can be ameliorated by using data from pilot studies or representative data from the literature. For example, a body of data from control animals could be used to estimate the population variation in metabolites anticipated to respond to a test chemical exposure. This again highlights the importance of collecting baseline data on age-matched and sex-matched control samples, introduced in Section 1.1. Best practice for estimating power and sample size will involve pilot or historical study data, combined with justification of the likely effect sizes from previous studies with a similar biological system and analytical technique. When pilot or historical study data are not available, simple traditional approaches (e.g. based on t-tests or linear regression) can be used, though the results should be treated with caution for the reasons stated above. In all univariate analyses, appropriate multiple testing corrections (e.g. Bonferroni or False Discovery Rate approaches) should be applied. In summarising power across many variables, the concepts of average power per variable or the proportion of variables reaching a desired power, are helpful. In many cases, an accurate calculation will depend on the specific regulatory question to be answered. For example, if dose-response curves are to be estimated, this modelling approach should be part of the power calculation.

Sample size estimation:

**REPORT**: source of population variation information; statistical approach to power and sample size estimation; minimal sample sizes to reach desired power thresholds.

### Randomisation and batching of samples

To ensure the validity of metabolomics data, considerable effort should be made to reduce analytical sources of error and bias. It is important to consider each stage in the process from sampling, through storage and preparation, to analysis. Best practice as a minimum will apply randomisation and blocking to sample preparation (including metabolite extraction) and data acquisition. For example, if samples must be extracted over a period of two days, equal numbers of randomly selected control and treated samples should be extracted on each day. Here, day is used as a blocking factor. Using randomisation and blocking, potential biases (e.g. varying extraction efficiencies, instrument drift, temperature fluctuations) can be minimized. Where samples are paired (e.g. before/after treatment), best practice would be to run each member of a pair consecutively, and to randomise the ordering of pairs. Where numbers of samples are small (<20), the analysis order should be checked to avoid accidental correlation with any experimental design factors.

When conducting larger studies that utilize hundreds or thousands of samples, it is recommended that sample processing and data acquisition be conducted in batches. Each batch should correspond to approximately equal numbers of samples from each experimental class (treatments and controls).

Randomisation and batching:

**REPORT**: Randomisation and blocking strategy used in each stage of sample preparation and analysis; number and size of batches.

## Supplementary Note 2. Analytical quality assurance and quality control

When conducting metabolomics experiments, it is essential to have a quality assurance framework as well as to use quality control samples to determine whether defined performance standards in a study meet the acceptance criteria. Quality assurance (QA) refers to all the steps that are conducted before an experiment and is defined as “a set of procedures that are done in advance of analysis and that are used to improve data” ^20^. This includes analyst training, ensuring that analytical instrument performance is maintained and certified, ensuring that standard operating procedures (SOPs) for sample collection, metabolite extraction, data acquisition, data processing and statistical analysis are developed and followed, and that any SOP deviations are tracked. QA also includes defining performance criteria. Quality Control (QC) is defined as “a set of activities that a laboratory does during or immediately after analysis that are meant to demonstrate the quality of project data” ^20,21^. Ideally, QA and QC is applied to cover all SOPs used in a metabolomics study, which include sample collection and processing, data collection, data processing, metabolite identification and statistical analysis. There needs to be documentation to describe all QA/QC procedures, as well as SOPs for all processes in the metabolomics study.

### Types of QC samples

In this section we first define and describe the five main types of QC samples focusing principally on their purpose(s) in a metabolomics study: system suitability QC, intrastudy QC, intralab QC, interlab QC and process blank (Supplementary Table 1). Following those descriptions, we provide additional guidance on what types of biological samples or metabolite standards can be used to make each of these types of QC samples (Supplementary Table 2). Finally, strategies for applying QC samples in a representative metabolomics study are described.

*Supplementary Table 1. Main types of QC samples, their purpose and considerations for their composition in metabolomics studies ^22,23^.*

| **Type of QC** | **Purpose of QC** | **Performance standards** | **Considerations for QC composition** |
| --- | --- | --- | --- |
| System suitability QC | To demonstrate analytical system is “fit for purpose” and working within specification with no contamination, prior to analysis of study samples. | Instrument responses to the system suitability QC are compared to thresholds for *m/z* shift, NMR chemical shift, chromatographic retention time shift, peak shape, and/or peak intensity to provide a go/no-go decision on whether study samples can be analysed. | Should be consistent over long periods and potentially usable across multiple laboratories. A synthetic sample comprising a mixture of authentic chemical standards or reference material can be used for this purpose. |
| Intrastudy QC (previously also known as ‘pooled QC sample’) | To (a) condition the analytical system, (b) provide measures of intrastudy reproducibility, (c) monitor, assess and potentially correct for systematic errors in measurements, e.g. drift in *m/z*, chemical shift, intensity and/or chromatographic retention time in QC samples (i.e. most often pooled samples) during a study and associated batches, to ensure that acceptance criteria are met for that specific study, and (d) optionally used to filter variables based on the linearity of their intensities in a dilution series of intrastudy QC (section 9.4). | Calculation of relative standard deviation (RSD; also known as coefficient of variation (CV)) of *m/z*, chemical shift, intensity, or chromatographic retention time provides quantitative assessment. Clustering in PCA provides qualitative assessment of similarity of intrastudy QCs. | Due to the nature of the algorithms applied to achieve these purposes, it is essential that the intrastudy QC is highly representative of the biological samples in the current study. It is practically derived from a small aliquot of all the individual biological samples within that study. These QCs are limited to one study. |
| Intralab QC (previously also known as a ‘Long-Term Reference (LTR) QC’) | To assess (and potentially correct for) any differences between separate studies within one laboratory. Hence this type of QC has also been referred to as an ‘Interstudy QC’. | Provides a measure of intra-laboratory analytical reproducibility over an extended time. | Should be representative of the study samples and hence derived from a one-time pool of multiple extracted samples by a specific laboratory using a defined SOP, or a synthetic sample covering the relevant metabolite space or a reference material of sufficiently similar metabolic composition to the study samples. |
| Interlab QC (previously also known as a ‘Long-Term Reference (LTR) QC’) | To assess (and potentially correct for) any differences between individual laboratories. | Provides a measure of inter-laboratory analytical reproducibility. | Should be accessible to multiple laboratories, has known providence, is stable, characterised and available in controlled batch numbers. Ideally this QC has a similar metabolic composition or matrix to the biological samples in the study, although this is not always possible, in which case use as close to the same composition as possible. |
| Process blank (previously also known as ‘blank’ or ‘extraction blank’) | To enable the measurement of interfering signals (‘contaminants’) that may arise from the ‘process’ - e.g. from extraction solvents, plastic ware, etc. - such that these contaminant signals can be removed from a study during the data processing. Sometimes used to assess carryover. |  | Study specific, prepared in the same manner as the biological samples except that no biological material is present. It is important to define the start and end points of the ‘process’ used to prepare this type of QC sample. |

### Sources of QC samples

A range of materials can be used to prepare each of these types of QCs, including from biological samples (commercial or specific to a laboratory) and single and mixtures of authentic chemical standards. Supplementary Table 2 summarises the options for sourcing each type of QC sample.

*Supplementary Table 2. Practical guidance on sourcing QC samples for metabolomics studies.*

| **Source** | **Type of QC** | **Notes** |
| --- | --- | --- |
| Pool of small aliquots of (typically all) extracted biological samples within *one* study. | Intrastudy QC | If biological samples in study are so small that a sub-aliquot cannot be taken, further (control) samples should be sourced and pooled to create intrastudy QC. Pooled QCs may be prepared in different ways and may or may not be dried down (or partitioned) before use. Whatever the case, the method of preparation for the pooled QC should be reported. |
| One-time pool of multiple extracted biological samples by a specific laboratory using a defined SOP; used across *multiple* studies. | Intralab QC, Interlab QC | If biological samples in study are so small that a sub-aliquot cannot be taken, further (control) samples should be sourced and pooled to create intrastudy QC. |
| Blank extraction | Process blank | Process (e.g. start and end points in the extraction protocol) must be defined. |
| Synthetic mixture of authentic chemical standards, prepared in-house. | System Suitability QC, Intralab QC | Synthetic mixture can be tailored for specific analyses and can be remade in-house as chemicals and concentrations are known. The metabolites in the synthetic mixture should span a range of physico-chemical properties that are similar to the study samples so that, for example for LC-MS, the authentic standards are distributed across the chromatographic retention time and *m/z* values. Authentic chemical standards can be isotopically labelled. |
| Standard Reference Material (SRM) sample | System Suitability QC, Intralab QC, Interlab QC | Certified quantitative levels of many metabolites are known and certified from a specific type of sample (<https://www.nist.gov/srm/srm-definitions>); e.g. NIST 1950 SRM is a plasma sample characterised for plasma metabolomics. |
| Reference Material (RM) sample | System Suitability QC, Intralab QC, Interlab QC | Equivalent to an SRM, except levels of metabolites are not certified (<https://www.nist.gov/srm/srm-definitions>). |

### Practical applications of QC samples

The practical application of QC samples in a metabolomics study has evolved over the past decade ^20,22–24^. Here we recommend current ‘best practices’, acknowledging that these will most likely continue to improve. First, System Suitability QCs should be analysed to ensure the analytical instrumentation is fit for purpose. If (standard) reference materials are used for the system suitability check, then these measurements could also serve as an interlab and/or intralab QC. Synthetic samples are most likely to be used as an initial system suitability check of analytical instruments due to their ability to be made consistently, in-house, over many years. It is recommended that extracted biological samples are not removed from their (short term) storage until the instrumentation has passed this test. Next, additional types of QCs and the extracted biological samples are loaded into plates / vials / tubes as appropriate for the instrumentation being used. Due to differences in the analytical capabilities and performance of NMR spectrometers versus mass spectrometers used in metabolomics, mass spectrometry studies require more extensive QC strategies. In the remainder of this section we introduce a QC strategy for LC-MS based metabolomics as an example.

The first samples to be analysed within the first batch of a study are ‘preconditioning’ samples (also referred to as ‘run-in’ or ‘dummy’ samples), which allow multiple components of the analytical system to equilibrate, e.g. chromatographic separation through to voltages in the detectors. Intrastudy QC samples are often used for these preconditioning samples, as they most effectively condition the instrumentation to samples representative of the biological samples. Process blank samples are typically measured at the start and end of each analytical batch. Intrastudy QC samples are analysed after every 5^th^ to 10^th^ biological study sample ^25^, to assess, and potentially correct, any drifts in measurement performance. Only small numbers of measurements of an intralab QC and interlab QC are needed within any one study.

System suitability QC: No community agreed acceptance criteria yet exist for this type of QC, however recommended criteria to apply to LC-MS metabolomics have been reported previously ^26^: (i) *m*/*z* accuracy better than the manufacturer’s listed instrument performance, (ii) retention time error of <2% compared to the defined retention time, (iii) peak area equal to a predefined acceptable peak area ± 10%, and (iv) symmetrical peak shape with no evidence of peak splitting. However, criteria should be tailored to laboratory specific requirements for each analytical assay ^22,25^.

**REPORT**: Source of system suitability QC sample and how it was prepared and stored; preparation date; performance achieved relative to acceptance criteria to indicate instrumentation is fit for purpose.

Intrastudy QC:

**REPORT**: Source of intrastudy QC sample and how it was prepared and stored; preparation date; measure of study analytical precision (study reproducibility).

Intralab QC:

**REPORT**: If used, source of intralab QC sample and how it was prepared and stored; preparation date; measure of laboratory analytical precision (laboratory reproducibility).

Interlab QC:

**REPORT**: If used, source of interlab QC sample and how it was prepared and stored; preparation date; performance standard achieved relative to specified amounts of metabolites (laboratory to laboratory reproducibility).

Process blank:

**REPORT**: Start and end points of the ‘process’ used to prepare this QC sample; peaks detected in process blank; whether peaks are removed from study dataset and threshold settings for their removal (see section 9.3).

## Supplementary Note 3. Sampling and metabolite extraction

Sampling and metabolite extraction are the next steps in the metabolomics workflow. While the specific protocols will depend on selecting the appropriate samples for the aim of the regulatory study (e.g. urine is usually preferred for studying the biotransformation of toxicants), the total number and physical form of those samples (i.e. liquid biofluid, solid tissue, etc.), the sample matrix, and/or the metabolites of interest, there are a series of generic steps to be followed for the majority of sample types: these include washing, quenching, potential storage, metabolic extraction, sample reconstitution in solvent for analysis, and the use of QC samples, each of which are described below. The most relevant samples for regulatory toxicology include urine, serum/plasma, dried blood spots, cells, cell media and tissue/whole organisms. A general sampling and metabolite extraction workflow is depicted in Supplementary Figure 1. Multiple publications have described metabolite extraction workflows, examples reported in *Nature Protocols* ^25,27^. All sample handling and extraction methods should follow documented standard operating procedures.


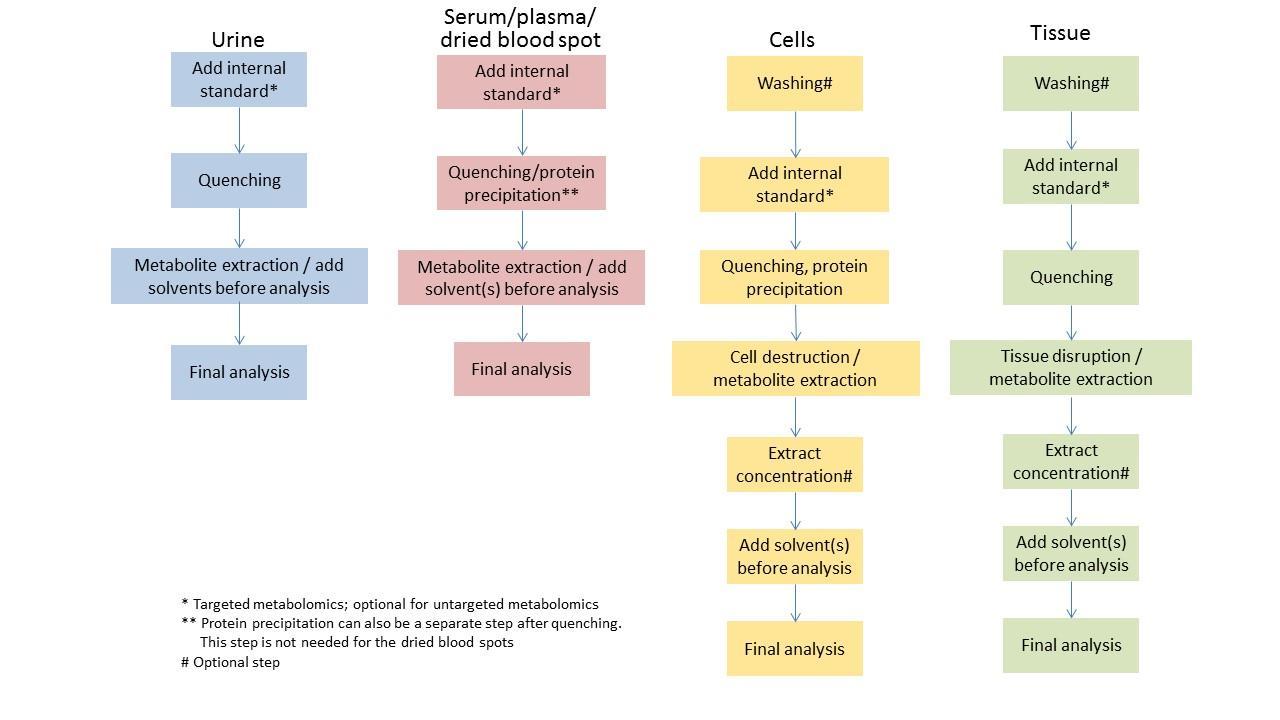
*Supplementary Figure 1: Schematic workflow for sampling and metabolite extraction from a range of common sample types.*

### Washing

The primary purpose of washing is to remove the contamination of one type of metabolome by a second type. For example, prior to the extraction of adherent cells (to study the cellular endometabolome), it is important to remove metabolites present in the cell media (which also contains the cellular exometabolome). For cells in suspension, it is best practice to either rapidly centrifuge or rapidly filter the material to separate the cells from the spent media, including wash steps ^28^. Tissues should be rapidly washed in saline to minimise contamination from the blood metabolome. Typically, these wash steps are immediately followed by quenching, described below. It is important to note that washing of cells or tissue can result in alterations of the metabolome being analysed, hence the use of untreated control samples that are processed using an identical protocol is essential.

**REPORT**: Wash solvent(s); washing procedure.

### Quenching

Best practice is to quench metabolism as soon as practically possible after sampling in a consistent manner (with a consistent washing protocol, if used). For adherent cells, quenching is typically performed by adding an ice cold solvent such as methanol, methanol:water, acetonitrile, acetonitrile:water or mixtures of these (sometimes acidified with formic acid). For cells in suspension, centrifugation or filtration is performed first to isolate the cells from the media, and the cell pellet is then directly snap frozen using liquid nitrogen. Similarly, for tissues or small whole organisms (e.g. zebrafish embryos), quenching is performed by snap freezing the sample in liquid nitrogen (or equivalent low temperature). For examples of cell quenching and extraction protocols, see those reported by ^29,30,31,28^. It should be acknowledged that quenching can never be perfect and that the levels of some rapidly metabolised metabolites (e.g. ATP) in the final sample will be quite different from those in the living system.

**REPORT**: Quench solvent; quenching procedure.

### Sample stability

Transport and storage conditions prior to metabolite extraction are important factors in the reliability of metabolite measurements. Storage temperature, time, and number of freeze-thaw cycles are significant factors that are related to the stability of endogenous metabolites, but also to the stability of the exposure chemical and potential biotransformation products formed. Short-term stability studies of plasma and serum samples have shown that significant changes in metabolite levels can occur at room temperature compared to -80 °C ^32,33^. Long-term stability studies of human plasma indicate that stability is metabolite specific ^34^, time, and temperature dependent ^35^. Fewer studies have been conducted on the stability of metabolites in urine, but pre-analytical sample handling and storage conditions have also been shown to be important factors ^36^. The number of freeze-thaw cycles of the sample can influence the stability of certain metabolites ^37^. Metabolite stability is also matrix dependent as freeze-thaw cycle studies of organ and plasma samples showed differences in stability (6). Best practice, after quenching, is to store the samples at low temperature (e.g. -80°C) and keep the freeze–thaw cycles to a minimum, including during the transportation phase. If samples are stored for a long period, it is recommended to study the impact of this on the stability of the metabolites of interest.

**REPORT**: post-collection handling, transportation method, storage temperature and duration, number of freeze-thaw cycles.

### Metabolite extraction

#### Extraction of urine, serum/plasma, dried blood spots, cell media

For liquid samples, metabolite extraction typically comprises the addition of a solvent (e.g. methanol, chloroform, or acid/base) to denature any proteins present and stop enzymatic activity (that will otherwise change the metabolome). This step, in practice, is often part of the quenching step (see for example, ^28–30^). For dried blood spots, a solvent is added to a punch of a dried blood spot to extract the metabolites ^38,39^.

**REPORT**: Extraction method SOP, including solvent(s), extraction temperatures and times, and potential storage temperature and duration, etc.

#### Extraction of cells

For some cell types with relatively fragile cell membranes, the quenching and extraction described above for liquid samples is sufficient to lyse cells, denature the proteins, and extract the metabolites into an appropriate solution. Some cell types have a strong cell wall and require a more aggressive treatment. During method optimisation it should be checked if cells are lysed after adding an extraction solvent using - for example - microscopy. If cells are intact an additional destruction step is needed, e.g. ultrasound agitation ^29^.

**REPORT**: Extraction method SOP, including solvent(s), extraction temperatures and times, and potential storage temperature and duration, etc.

#### Extraction of tissues

For tissue samples, the next step after quenching is the extraction of the metabolites from the matrix. Best practice for tissue extraction is the addition of an ice-cold organic solvent to the sample directly followed by destruction of the tissue. Another option is to lyophilize the samples before solvent extraction. Depending on the sample type, various types of disruption are available, such as ultrasound assisted extraction or bead-based homogenisation (e.g. ^27,40,41^). Best practice is to use an extraction method that does not show carryover of sample from earlier extractions, hence grinding tissue samples in a mortar is not recommended unless thorough cleaning between each sample is performed. For untargeted metabolomics, a general extraction procedure is recommended as a broad range of different types of metabolites from polar to non-polar metabolites (e.g. lipids and certain vitamins) should be extracted. A combination of solvents such as methanol:chloroform:water or methanol:MTBE:water are widely used for such extractions ^27,40,41^. After addition of the solvents and tissue disruption, the metabolites can be separated into polar (e.g. methanol/water) and non-polar (e.g. chloroform) fractions by addition of further solvent(s) (biphasic partitioning). If only polar metabolites are needed, extraction solvents include acetonitrile, methanol, or perchloric acid. To extract only non-polar compounds such as lipids, a methanol:chloroform solvent system can be used. For targeted metabolomics, the extraction solvent should be optimized to yield high extraction efficiencies and reproducible results. In all cases, best practice is to use extraction solvents of GC-MS, LC-MS, or NMR grade.

**REPORT:** Extraction method SOP, including solvent(s), extraction temperatures and times, and potential storage temperature and duration, etc.

### Extract concentration and reconstitution in solvent for final analysis

For most sample types (serum/plasma, cells, tissues) that have been extracted as above, the metabolite extract is often transferred to another solvent before it can be analysed. This requires the extract to be evaporated to dryness, typically using a vacuum centrifuge (if removing organic or aqueous organic solvents) or a freeze dryer (if removing only an aqueous solution). Most dried samples can be stored at low temperature (-80 ^o^C) until required for analysis. Finally, solvent(s) suitable for the data acquisition instrument are added as described in sections 4.1, 5.1, and 6.1. Solvents should be suitable for the instrument type. This drying and reconstitution step can also be used to concentrate samples. Urine and cell media are typically extracted and analysed directly without drying and reconstitution.

**REPORT:** Drying method; final reconstitution solvent(s), final volume, storage temperature and duration of extracts.

### Addition of internal standard

In targeted metabolomics, internal standards (IS) should be used to assess the accuracy and precision of metabolite concentrations (for example, Lewis et al., (2016), see also section 8.2). Internal standards are compounds that are added at the start of the sample treatment (e.g. the quenching step) in similar amounts to the level(s) of the metabolite(s) in the samples. Ideally, the internal standard should be added to the matrix prior to the quenching and/or extraction steps to correct for recovery losses. In general, ^13^C or deuterium labelled compounds are used as IS, or alternatively a compound that is stable, not naturally occurring (or never found in samples relevant to that study) and has a structure similar to the metabolite(s) of interest can be used. The IS corrects for possible recovery losses due to sample treatment and response variations of the instrumentation (e.g. ion suppression from matrix effects with mass spectrometry, see Section 5.3) during the sequence of sample analysis. In untargeted metabolomics, internal standards are optional and not widely used. The IS can provide information on the extraction efficiency of each sample. As opposed to targeted metabolomics, correction for the internal standard response between samples is normally not performed in untargeted analyses and only used as quality control measure.

**REPORT:** Internal standard(s); concentration internal standard.

### QC samples

Several types of QC samples, as described in section 2, should be prepared at this stage of the metabolomics workflow - in particular the process blank and intrastudy QC, which are both study-specific (i.e. prepared in each independent study). The intrastudy QC sample is formed by pooling small amounts (i.e. 10 to 50 μl) from every biological sample or sample extract so that sufficient QC volume is obtained for (i) use as preconditioning samples (for mass spectrometry analyses) and (ii) repeated analysis throughout the analytical batch (all analytical methods, though the intrastudy QC experimental design is method-dependent). The intrastudy QC samples should be stored, processed and analysed using the same SOP as the study samples. Synthetic mixtures can also be (re)prepared at this stage of the workflow, typically a subset of metabolites (e.g. 25 to 100 authentic chemical standards) that are present in the study samples.

**REPORT:** As described in section 2 (Quality assurance and quality control samples).

## Supplementary Note 4. Data acquisition and processing: Untargeted NMR

### Sample preparation and QC: NMR specific procedures

Prior to NMR data acquisition, an appropriate NMR solvent should be added to the extracted samples. Aqueous solvents (e.g. deuterium oxide) should be pH buffered with sodium phosphate and a chemical shift reference standard (e.g. sodium 2,2-dimethyl-2-silapentane-5-sulfonate-d6 (DSS)) added. When using volatile solvents (e.g. deuterated methanol) every effort should be made to reduce solvent loss so as to avoid the potential introduction of artificial inconsistencies in metabolite concentrations across samples; additionally, a chemical shift reference standard (e.g. tetramethylsilane) should be included. Prepared samples should be loaded into the appropriate NMR tubes or wells and cooled (4 ^o^C) until analysis.

Due to the high technical accuracy and precision attainable by NMR spectrometers, QA/QC procedures are less elaborate than those required for mass spectrometry, however the procedures are equally critical. For NMR, it is recommended that the system suitability checks performed each day include a full instrument set up and calibration, according to the protocol described in ^42^. This includes temperature calibration (using a deuterated methanol sample), water suppression assessment and if needed adjusting the centre of the spectral window to improve water suppression (using a sucrose sample), and the measurement of an external reference material for subsequent quantification purposes (e.g. Bruker QuantRef). Following this instrument set up, during the analysis of each analytical batch of study samples, it is recommended that a few intrastudy QC, intralab QC and process blank samples are analysed. Unlike for LC-MS, repeated analysis of an intrastudy QC every 5-10 study samples is not required, though their inclusion at a lower frequency (e.g. 1 every 100 samples) is useful for large studies.

**REPORT:** Sample concentration and reconstitution solvent for final analysis (as described in section 3.4).

**REPORT:** QA/QC procedures for NMR instrument set up and calibration/tuning; QA/QC procedures used during analysis of study samples (as described in section 2).

**REPORT**: acquisition order of all QC samples and study samples.

### Data acquisition: NMR

Collection of one dimensional (1D) spectra in an untargeted fashion (i.e. no preselection of metabolites) is the primary means by which samples are analysed using NMR spectroscopy in metabolomics studies. However, a variety of user-defined parameters are employed during the acquisition of these spectra which, if not consistent across studies, can have large impacts on the quality, reproducibility, and interpretability of the results. While, it is recognized that some degree of flexibility is necessary to accommodate the different sample types that may ultimately be used for metabolomics-based regulatory assessments, the establishment of boundaries and recommendations for data acquisition will serve to establish an acceptable level of quality and rigor that are necessary to instil confidence in the validity of results. Ideally, best practices for data acquisition should include those that both provide high quality and reproducible spectra and are widely accepted within the international community. The guidelines recommended below for acquisition of 1D ^1^H-NMR spectra meet both of these criteria.

Sweep width: A sweep width of 12 ppm is necessary to best capture the range of metabolite resonances present in biological samples.

**REPORT**: Sweep width value.

Carrier frequency: The transmitter should be set to a carrier frequency matching that of the water resonance.

**REPORT**: Carrier frequency used and frequency of water resonance.

Pulse width: The pulse width for a 90 degree pulse should be less than 10 microseconds. The requirement for pulse widths greater than 10 microseconds may be an indication that the tuning and/or matching of the NMR detector (probe) has not been properly conducted.

**REPORT:** Time (in microseconds) used to apply 90 degree pulse width.

Predelay: Set to no less than 2 s (including saturation time).

**REPORT:** Pre-delay time.

Acquisition time: Set to no less than 1 s.

**REPORT:** Acquisition time.

Temperature: Sample temperature should be maintained at a constant value between 20 ^o^C to 30 ^o^C (293 K to 303 K).

**REPORT:** Sample temperature.

Optional: Although their reporting is not required, we also recommend the following best practices to ensure that valid 1D ^1^H-NMR spectra are collected:

**REPORT:** Lock frequency matches that of the deuterated solvent used to solubilize extracted metabolites.

**REPORT:** Lock phase optimized to maximize the lock level.

**REPORT:** Probe temperature variation of less than 0.1 ^o^C.

**REPORT:** Sample spinning was not used.

### Data Processing: NMR

In common with other platforms, the version and source of software must be reported for all analysis steps. This includes settings of important parameters, particularly those which have been modified from default values.

**REPORT:** Software source, version and parameters.

#### Preprocessing: NMR

This section covers the process of converting raw NMR data acquired on an instrument to processed NMR data which would be considered by an analytical chemist to be of sufficient quality for manual interpretation, i.e. conversion to a series of individual NMR spectra, one per biological sample. Typically only 1D NMR data are acquired on every sample, with 2D data acquired primarily for metabolite identification purposes (with the potential exception of 2D *J*-resolved NMR spectroscopy which is gaining in popularity as a high throughput NMR metabolomics method in research projects ^43^). Hence, here we focus on best practice and reporting for 1D data preprocessing. In comparison to mass spectrometry metabolomics datasets, the preprocessing steps for NMR are relatively straightforward. At a minimum, these include (1) application of an apodization function, (2, 3) phase and baseline correction, and (4) chemical shift calibration. These steps are essential for the preprocessing of raw NMR spectra prior to subsequent metabolomic data analyses and thus included here as best practices. Other aspects of preprocessing such as zero filling and linear prediction are often employed, and should be reported when used, but are not essential for all datasets and therefore considered optional.

Apodization: An exponential weighting function ≤ 0.3 Hz is recommended.

**REPORT**: Window function type; window function magnitude.

Phase correction: Manual or automatic methods are acceptable.

**REPORT**: Phasing method; phasing parameters.

Baseline correction: Low-order methods are recommended, such as constant, linear, or polynomial.

**REPORT**: Baseline correction method; parameter optimisation.

Chemical shift calibration: Manual or automatic methods are acceptable.

**REPORT**: Method used for chemical shift calibration.

Assessment of spectral quality: An assessment of the general quality of all spectra is recommended. At a minimum, the chemical shift reference peak width at half height should be reported. However, recognizing that assessments of spectra quality are frequently sample and study specific, and hence hard to set widely applicable quantitative values for, we also recommend submission of a .pdf file containing an image(s) of aligned and stacked spectra for visual inspection.

**REPORT**: Chemical shift reference peak width at half height (for every spectrum); submission of .pdf file containing an image(s) of all aligned and stacked spectra.

#### Data reduction: NMR

This section covers the process of converting preprocessed spectral data into a tabular form for statistical analysis. Best practice includes (1) an optional peak alignment and matching step, followed by either (2) use of full resolution NMR spectra, (3) binning, and/or (4) peak fitting.

Alignment: (optional) forms a correspondence between peaks in different samples which may exhibit small shifts. To preserve peak areas, linear correction methods are considered best practice.

**REPORT:** Alignment method; alignment parameters.

Full resolution: No data reduction is performed. Spectra are formed into an intensity matrix where each row is a sample and each column is a chemical shift (ppm).

**REPORT:** Full resolution used.

Binning: Either a uniform or non-uniform bin grid may be used, with automatic or manual placement of bins. Each bin is summarised by its total integrated intensity.

**REPORT:** Binning method; binning parameters.

Peak fitting: Peaks are first detected and then fit to a library of standard compound spectra. The fit generates a value quantifying the relative concentration of each compound.

**REPORT:** Peak fitting method; peak fitting parameters.

## Supplementary Note 5. Data acquisition and processing: Untargeted LC-MS

### Sample preparation and QC: LC-MS specific procedures

Prior to data acquisition, samples should be in solvents that facilitate their ionization and are compatible with the LC mobile and stationary phases ^25,44^. Samples should be randomised into analytical batches as introduced in section 1.5. Studies should rigorously adhere to QA/QC procedures due to the levels of technical variability inherent to mass spectrometry analyses.

For LC-MS it is recommended that a system suitability QC is measured to ensure that chromatographic retention time, *m/z* measurements and peak intensities are within specification. It is essential that the measurement of study samples is preceded by several ‘preconditioning’ samples (best practice is to use intrastudy QC samples) to allow the LC and MS to equilibrate, and process blank QC samples to provide a measure of background contamination ^25^. Intrastudy QC samples should be analysed every 5 to 10 biological samples, providing extensive valuable measurements for assessing and potentially correcting any drifts in measurement performance ^22^. In addition, intralab QC and interlab QC samples should be used within any one study.

**REPORT:** Sample concentration and reconstitution in solvent for final analysis (as described in section 3.4).
**REPORT:** QA/QC procedures used for LC-MS instrument set up, calibration and tuning (introduced in section 2); QA/QC procedures used during analysis of study samples (as described in section 2).
**REPORT:** Acquisition order of all QC samples and study samples.

### Data Acquisition: LC-MS

For untargeted metabolomics, no internationally standardized data acquisition methods are available, although a number of well-tested protocols are now in place; see for example *Nature Protocols ^25,44^*. Additionally, some metabolomics facilities have agreed to apply the same analytical methods for untargeted methods in order to increase the consistency of data acquisition, e.g. the Phenome Centres that are distributed across several countries. As the goal of untargeted LC-MS metabolomics is to reliably detect as many metabolites as possible, it has become widely accepted and therefore recommended to use complementary LC columns, such as a HILIC and C18, to characterise both polar and non-polar metabolites. It is also recommended to analyse samples in both positive and negative ionization modes to maximize coverage of the metabolome.

Liquid chromatography instrument configuration

**REPORT:** LC manufacturer, model number, software package(s) and version number(s); LC column and pre/guard column manufacturer, model number/name, stationary phase composition and particle size, internal diameter, and length; injection vials and plates manufacturer and model number.

Liquid chromatography method

**REPORT:** LC method name, mobile phase compositions, mobile phase flow rate, composition of the wash solvent, column temperature and pressure, gradient profile, and amount of sample injected.

Mass spectrometry instrument configuration

**REPORT:** MS manufacturer, model number, software package and version number; ionization source (ESI, APCI, APPI, etc.), source voltage, source temperature and gas flows; type of mass analyser (Orbitrap, time-of-flight, FT-ICR, ion-trap, etc.).

Mass spectrometry method

**REPORT:** Acquisition mode (full scan, MSn, etc.); polarity (positive or negative ion analysis); *m/z* scan range; mass resolution; lock spray (optional).

When RAW data has been collected in “profile” mode (where a peak is represented by a collection of signals over several scans), it is recommended that data should also be centroided (in centroid mode, the signals are displayed as individual *m/z* with zero line widths). It is recommended to use vendor-specific algorithms to centroid mass spectra. Centroiding algorithms are available via open source solutions, such as *msconvert* (*Proteowizard library and tools)* or via vendor software. Centroiding mass spectra is usually included as one of the processing steps when vendor files are converted into open-format files.

**REPORT:** Software and algorithm for centroiding (optional).

### Matrix effects

LC-MS(/MS) suffers from an important drawback, which is its susceptibility to matrix constituents. To obtain the best quality of signal, features should be selected with respect to intensity, retention time, peak shape and matrix effect. Matrix effects, mostly related to electrospray ionisation sources, can be estimated by either post-column infusion for qualitative estimation ^45^ or by the method described by Matuszewski et al. for quantitative estimation ^46^, after selective sample preparation. Describing the study of matrix effects is now required in some regulatory documents, such as by the US Food and Drug Administration, which recommend a strict evaluation of the matrix effects during the use of LC-MS(/MS). Matrix effects can not only be detrimental to accurate quantification but also to multivariate data analysis of untargeted LC-MS metabolomics. In most toxicological studies, semi-quantitative analyses are conducted on nearly identical biological matrices, therefore matrix effects are often considered similar for all the samples within a study. When using chromatography, a variable selection could be made according to the retention factor (k’) of the observed features. A high k’ should present the lowest interfering issues according to the fact that the chromatographic selectivity is higher.
 **REPORT:** Method to assess matrix effects (optional).

### Data Processing: LC-MS

In common with other platforms, the version and source of software must be reported for all analysis steps. This includes settings of important parameters, particularly those which have been modified from default values.

**REPORT:** Software source, version and parameters.

#### Data preprocessing: LC-MS

Typically, raw data obtained from the instrument software does not require further preprocessing to be interpretable (but see data reduction below). Some of these operations may optionally be performed: baseline correction, noise reduction and smoothing may be performed.

Baseline correction (optional)

**REPORT:** Baseline correction software; algorithm; parameters.

Noise reduction and smoothing (optional)

**REPORT:** Noise reduction software; algorithm; parameters.

#### Data reduction: LC-MS

This involves four steps: (1) peak detection/picking, (2) retention time alignment, (3) grouping/matching, and (4) peak integration.

Peak detection/picking: Best practice methods take account of the much higher resolution of the *m/z* dimension than the retention time dimension, e.g. centWave from XCMS.

**REPORT:** Peak detection/peak picking software; algorithm; parameters.

Retention time alignment: This is necessary to take into account shifts in retention time of the same analytes in different samples. Care should be taken to avoid over-correction. Methods based on dynamic time warping (e.g. OBI-Warp in XCMS) are considered best practice.

**REPORT:** Retention time alignment software; algorithm; parameters.

Grouping/matching: Peaks from the same analyte in different samples must be matched. Best practice methods allow for non-detection of analytes in some treatment groups (e.g. controls). Further round(s) of retention time alignment and grouping/matching may be iterated to improve data quality.

**REPORT:** Grouping/matching software; algorithm; parameters.

Peak integration: Estimation of the relative peak abundance normally involves integration of the area under the extracted ion chromatogram in each sample. Use of peak height as an abundance measure is optional but should be justified (e.g. in cases of peak crowding, and justified by showing a linear response to increasing metabolite concentration).

**REPORT:** Quantification software; algorithm; parameters.

## Supplementary Note 6. Data acquisition and processing: Untargeted GC-MS

### Sample preparation and QC: GC-MS specific procedures

Metabolite extraction for GC-MS is dependent on the class (or classes) of compounds being analysed and the biological system from which they are being extracted. In general, there is an extraction step and, if required, a clean-up step to remove matrix/protein or, more commonly, separate polar from non-polar compounds. Often one or more derivatization step(s) of the polar and non-polar fractions is required to make the compounds suitable for GC-MS analysis. QC considerations are largely the same as for LC-MS, with some slight alterations. For example, fewer run-in samples are required before the sample sequence (generally 3-6 are used), while retention time shifts are largely negligible over the course of a sample sequence (although this should always be checked by the operator, especially on sequences of greater than 50 samples). The reader should refer to current literature for validated methods and QCs for their particular study (e.g. ^25,47–49^).

**REPORT:** Derivatization reagents and reaction (including incubation temperature and time); clean-up/partitioning (if used).

**REPORT:** Sample concentration and reconstitution solvent for final analysis (as described in section 3.4); QA/QC procedures used for GC-MS instrument set up, calibration and tuning; QA/QC procedures used during analysis of study samples (as described in section 2).
**REPORT:** acquisition order of all QC samples and study samples.

### Data Acquisition: GC-MS

The acquisition of GC-MS metabolomics data should follow recommended protocols, such as published in *Nature Protocols* and relevant book chapters ^25,47–49^.

Gas chromatography instrument configuration

**REPORT:** GC manufacturer, model number, software package and version number; GC column manufacturer, model number/name, stationary phase composition, internal diameter and length; manufacturer and model number of injection vials.

Gas chromatography method

**REPORT:** GC method name, inlet system (e.g. split/splitless), inlet temperature (including whether constant or ramped), transfer line temperature, gas flows and pressure, temperature gradient, amount of sample injected.

Mass spectrometry instrument configuration

**REPORT:** As for section 5.2.

Mass spectrometry method

**REPORT:**  As for section 5.2.

### Data Processing: GC-MS

In common with other platforms, the version and source of software must be reported for all analysis steps. This includes settings of important parameters, particularly those which have been modified from default values.

**REPORT:** Software source, version and parameters.

#### Data preprocessing: GC-MS

Typically, raw data obtained from the instrument software does not require further preprocessing to be interpretable (but see data reduction below). Some additional operations may optionally be performed, such as baseline correction, noise reduction (e.g. SIM), and smoothing.

**REPORT:** Baseline correction method; noise reduction method; smoothing method (if performed).

#### Data reduction: GC-MS

The process of data reduction is much the same as for LC-MS, but with emphasis on different steps. It can be argued that, at the time of going to press, the range and sophistication of GC-MS data analytical tools is not to the level of LC-MS equivalents. With that in mind, data processing is perhaps more commonly performed vendor-specific software than LC-MS, although a number of alternative software packages exist, each with their own specialities (for example, *AMDIS* and *GAVIN*, developed especially for GC-MS, and ‘GC-MS capable’ versions of LC-MS packages, e.g. mzMatch, IDEOM, and AnalyzerPro).

Peak detection/picking: GC-MS mass resolution is often much lower than LC-MS, largely due to the popularity of classic, single quadrupole MSD detectors. However, GC chromatographic resolution is often much better, and more robust than LC (see below). In most (but not all) cases, chromatographic peaks consist of fragment ions of only one molecule (although co-elution of a few molecules at a time does occur). Hence peak picking and deconvolution is simpler than for LC-MS. In its simplest terms peak picking and deconvolution for individual molecules is performed by determination of the local minima of the largest (most abundant) of the co-aligned ions for each (putative) molecule.

**REPORT:** Peak detection/peak picking software, algorithm and parameters.

Retention time alignment: GC chromatographic resolution is much more robust than LC, largely due to the reproducibility of the mobile phase (commonly helium) and the resistance to contamination by particulate matter (which often gets trapped by the inlet). Due to this, retention time drift is often not an issue within a GC-MS sample run, although it can happen over longer (>50 samples) runs and between runs. In these cases, as for LC-MS, retention time alignment is necessary.

For GC-MS, retention times can be converted into instrument independent retention indices (RI). Use of RI values rather than retention times, can alleviate the need for retention time alignment. RI values are entirely dependent upon the congruence of all data acquisition parameters (derivatization agent, column chemistry, oven temperature gradient, inlet temperature, pressures, flows, etc.) and this should be considered when comparing inter-experiment RI values.

**REPORT:** Either retention time values (and associated alignment software, algorithm, and parameters), or RI values (and calculation method, including standards used and whether non-linear or linear regression modelled), as appropriate.

Grouping/matching: Again, this is often less of a concern than in LC-MS, although the basic principles still apply. Best practice methods use at least one validation ion (an ion semi-specific for that molecule that can be used to confirm the annotation/identification of the molecule suggested by the quantifier (main) ion.

**REPORT:** Grouping/matching software, algorithm and parameters.

Peak integration: As for LC-MS, above.

**REPORT:** Quantification software, algorithm and parameters. Use of internal and external standards for quantification and identification.

## Supplementary Note 7. Data acquisition and processing: Untargeted DIMS

### Sample preparation and QC: DIMS specific procedures

Best practice includes resuspending samples in appropriate solvents to facilitate the ionization of metabolites, for example: for positive ionization mode analysis of polar metabolites, methanol:water:formic acid; for negative ion polar metabolomics, methanol:water:ammonium acetate; and for positive and negative ion lipidomics, methanol:ammonium acetate:chloroform and any modifiers added to facilitate ionization (with ammonium or lithium salt modifiers).

Randomisation and batching of samples, as well as the QC procedures for untargeted DIMS, largely follow those as described for LC-MS in section 5.1, except that QC procedures related to chromatographic retention time should be ignored. Also, fewer ‘preconditioning’ intrastudy QC samples are required for DIMS as only the MS is required to equilibrate.

**REPORT:** Sample concentration and reconstitution in solvent for final analysis (as described in section 3.4).

**REPORT:** QA/QC procedures used for DIMS instrument set up, calibration and tuning (introduced in section 2); QA/QC procedures used during analysis of study samples (as described in section 2).

**REPORT:** acquisition order of all QC samples and study samples.

### Data Acquisition: DIMS

Best practice for high sensitivity DIMS-based polar metabolomics and lipidomics has been described in *Nature Protocols ^50,51^*. This particular method utilizes nanoelectrospray ionization (nESI), which minimizes ionization suppression or enhancement effects as compared with standard electrospray ionization. DIMS data is often recorded as several overlapping mass-to-charge (*m/z*) windows that are subsequently ‘stitched’ together to create a complete mass spectrum (termed spectral stitching), which considerably increases the dynamic range and detection sensitivity. The data acquisition is reproducible, rapid and automated, which enables analyses of >10,000 samples per year per instrument.

Mass spectrometry instrument configuration

**REPORT:** As for section 5.2.

Mass spectrometry method

**REPORT:** As for section 5.2.

### Data Processing: DIMS

In common with other platforms, the version and source of software must be reported for all analysis steps. This includes settings of important parameters, particularly those which have been modified from default values.

**REPORT:** Software source, version and parameters.

#### Data preprocessing: DIMS

As for other MS methods, DIMS spectra should be screened to identify any electrospray failures during acquisition. Failures should be removed from the dataset for further processing.

**REPORT:** Samples excluded from further data processing and analysis.

#### Data reduction: DIMS

Mass calibration: High mass accuracy and precision are essential for accurate processing as well as metabolite annotation, and, therefore, internal calibration is the preferred step for minimizing mass uncertainty. Internal calibration is achieved by using the accurate masses of metabolites known to be present in the mass spectra of the biological samples. Note the same principle applies to GC-MS analysis, but is referred to as internal ‘tuning’. External calibration - using a QC sample - is also acceptable.

**REPORT:** Algorithm and parameters used for internal *m/z* calibration.

Noise filtering: A two-stage signal filter can be used to discriminate genuine metabolite signals from noise. This approach requires that each sample is analysed multiple times. The first stage of filtering comprises a hard signal-to-noise ratio (SNR) threshold, which is followed by a replicate filter. Only peaks with a SNR value above the defined threshold should be considered real and retained in the dataset. The replicate filter requires that for a peak to be deemed real it must be present in at least x-of-the-n replicate measurements.

**REPORT:** SNR threshold; replicate filter settings.

Peak alignment: The noise filtered mass spectra from multiple biological samples are combined into a single signal intensity matrix with samples as rows and *m/z* features as col­umns.

**REPORT:** *m/z* range (as ppm) for peak alignment.

## Supplementary Note 8. Data acquisition and processing: Targeted MS assays

### Targeted metabolite analysis

Targeted metabolite analysis has key criteria that differ from those required for untargeted metabolomics. For example, metabolite identification is essential, while metabolite recovery and quantification accuracy are of paramount importance. Therefore, metabolite extraction and data acquisition methods are used to maximize the recovery and subsequent detection of specific metabolites, often to the detriment of other metabolites deemed less important to that study. Statistical power considerations are also different to those for untargeted metabolomics and, as a general rule, fewer biological replicates are required in targeted studies.

**REPORT:** Data acquisition instrumentation and SOPs as for untargeted analysis (see above, sections 5-7).

### Use of reference standards in targeted metabolite analysis

The primary objective of targeted metabolite analysis is to accurately quantify one to many pre-selected, and confidently identified, metabolites. Given this, the use of single or mixtures of reference standards, either ‘internal’ or ‘external’ to the study samples, is essential for successful data analysis. Traditionally, these reference standards have mostly been made ‘in-house’ by individual laboratories and are largely defined by the specific metabolites or metabolic pathways of interest. More recently, commercial kits containing a panel of reference standards (internal and external) have become available, often in convenient multi-well plate formats. Indeed, some companies offer a commercial assay service, whereby samples can be sent from multiple laboratories to be analysed in a standardized manner. However, whatever reference standards are used, best practice in regulatory toxicology requires transparency of the approaches and consistent reporting standards. As such, identical reference standards should be available for all targeted MS assays within a study (both intra- and interlab), with mixtures ideally being prepared by/sourced from one single laboratory. Furthermore, the ability to report full reference standard content and associated methods for metabolite identification and quantification should be ensured before commencing a project.

**REPORT:** Identity(ies) and concentration(s) of reference standards; source of reference standards; whether reference standards are used internally or externally.

### Identification and Quantification

#### Identification

Current standards for metabolite identification are discussed in Section 11.

#### Absolute quantification

Absolute quantification is dependent on using appropriate reference standards within a sample batch. This can be achieved by:

**(a)** analyzing an external reference standard (comprising of a single metabolite or mixture, the latter usually containing ~50-400 metabolites) at regular intervals throughout the sample batch, and/or

**(b)** adding an internal reference standard (either a single metabolite or mixture) into every study sample.

Guidelines for the suitability of an internal standard are described in Section 2.

#### Relative quantification

Limits of quantification for each metabolite standard should be predetermined. Metabolites present in study samples outside of these limits may be ‘detected’ but not quantified. When authentic standards are not available (e.g. for all lipid species within a particular class), have not been analysed, or when metabolites are unidentified, relative quantification of metabolites should be reported.

No matter what MS technique is used, the reporting standards for quantification remain the same:

**REPORT:** Software/algorithm/calculation used.

### Considerations for targeted LC-MS

Due to analysis-to-analysis variability, even on the best-performing instruments, *absolute* quantification is often not realistically possible (or advisable) for many metabolites simultaneously, and so abundances are mostly reported as ‘relative’. The most appropriate method for calculating absolute abundances using LC-MS is by the inclusion of isotopically-labelled internal standards and the use of SRM (single reaction monitoring) or MRMs (multiple reaction monitoring, i.e. the selected monitoring of multiple product ions from one or more precursor ions) for every metabolite that is measured. Here, known amounts of an array of stably-labelled relevant molecules are spiked into each sample. Quantification can then be calculated by comparison of the peak areas of the labelled and unlabelled versions of each metabolite. This method also gives an additional level of confidence to metabolite identification.

Given the necessity of labelled versions of each molecule of interest to be included in each sample, such experiments rapidly become prohibitively expensive. Moreover, labelled versions of many molecules are not commercially available and are difficult, or impossible, to synthesise ‘in-house’. In these cases, targeted assays become reliant upon external reference standards and quantification should be referred to as *relative*.

### Considerations for targeted GC-MS

While quantification is challenging in LC-MS, the robustness of GC-MS chromatography and constant fragmentation voltage (when using electron impact ionization) give the user more confidence in *absolute* quantification of many metabolites. While internal reference standards can be added to each sample (as for LC-MS), it is not always deemed as necessary for GC-MS, due to the higher analysis-to-analysis reproducibility of the instrument. For GC-MS, it is acceptable to quantify metabolites by comparison to external reference materials (see section 8.3.2.), provided that each metabolite detected in both the reference standard and study sample lies within the dynamic range (within the limits of quantification) of that molecule, and that an appropriate internal standard(s) has been included in all samples and the external reference standard. When these conditions are met, the abundance for each chemical can be calculated:

*Absolute abundance = (peak area of chemical ÷ peak area of internal standard) x (amount of internal standard ÷ MRRF),* where *MRRF = (peak area of chemical standard ÷ peak area of internal standard), when the amounts of each are equal. The ‘MRRF’ is the ‘molar relative response factor’.*

As with LC-MS, where authentic reference standards are not available, have not been analysed, or when metabolites are unidentified, only *relative* quantification should be reported. When abundances lie outside the dynamic range, a feature may only be reported as ‘detected’ or ‘not detected’.

### Considerations for targeted DIMS

Direct infusion mass spectrometry is an approach that does not use any chromatography to aid the separation of metabolites, thereby reducing costs and analysis times. Targeted DIMS methods use MRMs (i.e. the selected monitoring of multiple product ions from one or more precursor ions) for every metabolite that is measured. There are several targeted DIMS kits on the market (e.g. Biocrates p180 and p400 HR kits) that provide quantitative data and use instrument specific SOPs. The Biocrates kits use blanks, internal standards, system suitability checks and quality control in low, medium and high concentrations. Even with this significant repertoire of QC samples, these kits have a warning that states “not to be used for diagnostics”. Nevertheless, the Biocrates kits have performed well in round-robin laboratory comparisons ^52^. The Sciex Lipidyzer platform uses direct infusion coupled to ion mobility to detect over 1100 lipids from 13 different classes. The Lipidyzer uses over 50 labelled internal standards, ion mobility tuning standards, system suitability standards, and control plasma samples with spiked standards at known concentrations to improve the quantitative accuracy, precision and reproducibility. The Sciex website states that the Lipidyzer performance capabilities has been validated across 3 laboratories.

## Supplementary Note 9. Data post-processing

This section covers remaining data processing steps required to transform the data into a form amenable to statistical analysis. To facilitate the reader’s understanding of where this fits in the workflow, Supplementary Figure 2 summarizes the entire data pre-processing, post-processing, and statistical analysis workflow. Data post-processing usually requires production of a 2-dimensional data table with each sample represented in one dimension (typically a row) and each metabolic variable (e.g. quantified peak) in the other dimension (typically a column). The entries in the data table indicate the (relative or absolute) quantity of the specific variable in each sample. The procedures shown in sections 9.1 to 9.4 below represent perhaps the most common order for these steps. However, the order of these procedures can be changed, depending on the data acquisition and processing approaches used.


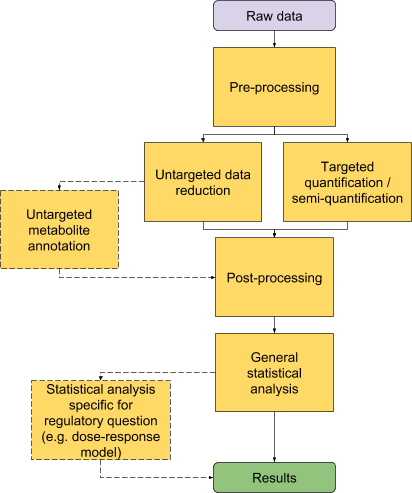


*Supplementary Figure 2: Data post-processing and statistical analysis workflow. Dotted lines indicate optional steps.*

In common with other stages of analysis, the version and source of software must be reported for all data processing steps. This includes settings of important parameters, particularly those which have been modified from default values.

**REPORT:** Software source, version and parameters.

### Signal intensity drift / batch correction

While every effort should be made experimentally to minimise variations in signal intensity within and between batches, these can be (partially) corrected in the data processing step, typically using signals recorded in the intrastudy QC samples. In some cases (e.g. small studies with NMR) these effects may be negligible and therefore the correction step is made optional. However, best practice should include an evaluation of the need for correction.

Presence of within- and/or between-batch signal intensity drift: Evaluation of the presence of such signal intensity drift typically includes a PCA analysis, showing both biological and intrastudy QC samples and RSD for some metabolites present in QC samples, (covering a wide range of physicochemical properties and concentrations). If the intrastudy QC samples show significant differences in scores or RSD values between batches, or a drift in scores values or RSD values within any batch, then the effects are present.

**REPORT:** PCA scores plots showing biological and intrastudy QC samples. Optionally, additional PCA of just the intrastudy QC samples, for example from multiple batches, provides a more effective evaluation for drift.

Batch correction (optional): Many algorithms exist for batch correction, for example scaling to the intrastudy QC. Currently there is no consensus on the best method. If a batch correction method is used, its effects should be demonstrated, for example with a PCA analysis and by inspecting the intensities of representative analytes, both before and after the batch correction.

**REPORT:**  Batch correction method and parameters; evidence that batch correction has been successful (e.g. PCA scores plots showing intrastudy QCs).

Signal intensity drift correction within a batch (optional): Many algorithms exist for signal intensity drift correction, for example scaling to the intrastudy QC. Again, there is no consensus on the best method. If a correction is made, its effects should be demonstrated, for example with a PCA analysis and by inspecting the intensities of representative analytes, both before and after the correction is made.

**REPORT:** Signal intensity drift correction method and parameters; evidence that the correction has been successful (e.g. PCA scores plots showing intrastudy QCs).

### Missing value imputation

Missing values occur in most metabolomics data sets, for a variety of reasons, such as loss of samples, failure to detect a peak in a given sample or data processing effects. Their presence can significantly affect the performance of the statistical analysis and thus influence the results of the study. Therefore they must be examined and reported carefully.

The biological, analytical or data processing reason for the presence of missing values should be identified if possible. The distribution of missing values should be analysed and recorded; e.g. are values missing at random? Are many values for specific samples or variables in the data set missing? The removal of variables and/or samples with large proportions of missing values is described in Sections 9.4 and 9.5, respectively. Detection of a peak in the control samples can be used as a measure of confidence in the data; this also allows fold changes to be calculated. Nonetheless, analysis is not restricted to peaks meeting this criterion; peaks not detected in controls can be flagged. Typically, even after the removal of particularly problematic variables and/or samples, a number of missing values will remain in the dataset. Missing value imputation can then be applied and reported ^53,54^.

Imputation (optional): Using zero or lowest recorded values as imputation value should be avoided in untargeted data sets. kNN imputation is recommended as best practice ^53^. For targeted assays, imputation with LOD or LOQ can be appropriate depending on the subsequent statistical analysis.

**REPORT**: Reasons for missing values (if known); any patterns in missing value distribution (e.g. in *m/z*, RT, intensity, presence/absence in control group); threshold for sample/variable exclusion; imputation method and parameters (if used).

### Normalisation

Normalisation is the process of removing technical or otherwise irrelevant variation from the data on a sample by sample basis. Typically, intensity data for each sample is multiplied by a scalar factor, which is different for each sample. Normalisation is usually applied to take account of uncontrolled factors such as dilution or overall instrument response.

The choice of normalisation must be justified. For untargeted metabolomics analysis in a toxicology study, for which the proportion of the metabolic phenotype changing between untreated and treated test samples is often relatively small, a preferred approach is probabilistic quotient normalisation (PQN). For targeted analysis, normalisation to an internal standard is recommended.

**REPORT**: Normalisation algorithm and parameters.

### Filtering of variables

In some cases, the analytical measurement procedure may result in significant numbers of noisy and/or systematically biased variables. For example, in untargeted LC-MS there may be many low intensity noisy features. It may be desirable to filter the data to remove such low-quality noisy features to improve the overall reliability of the data set. The extent to which filtering is acceptable will depend on the analytical method employed and the mode of analysis. In general, variables from a targeted analysis will be more reliable and require less filtering than those from an untargeted analysis.

Several different filtering processes may be conducted, all of which are optional.

Removal of variables that are sparsely detected across samples (optional): Features are removed if they are only present below a defined percentage of study samples; e.g. if a feature is present in <50% of samples then it is often removed. Care should be taken to ensure the missing values are distributed across several of the study groups in order to avoid removing real features; e.g., a real feature may be mostly present in one group exposed to a chemical but not present in the control group, where this real feature is an endogenous metabolite that significantly increases in intensity upon chemical exposure. An alternative variable filtering strategy is to retain peaks if they are present in a given percentage of any one group.

**REPORT:** Method for removing sparsely detected variables, including threshold, if used.

Filtering for repeatability (optional): A widely used procedure in untargeted LC-MS and DIMS is to remove features with high analytical variability in intensity. The RSD of the intensity of each feature can be estimated from the intrastudy QC samples and features with an RSD greater than a threshold (typically 30%) are removed.

**REPORT:** Method for repeatability filtering, including RSD threshold if, used.

Filtering for linearity (optional): Some analytical designs may include a dilution series, where an intrastudy QC is diluted by known factors (e.g. 2, 4, 8, 16, etc.) and analysed with the other samples. Within the instruments linear range, reliable variables should exhibit intensities which correlate very strongly with the known dilution factors. A feasible strategy is therefore to remove variables whose intensities do not correlate well to the dilution factors (e.g. using Pearson correlation < 0.8).

**REPORT:** Method for linearity filtering, including correlation threshold, if used.

Removal of uninformative features (optional): Features which do not change across the study are unlikely to be informative on toxicology and can often be removed. This can facilitate statistical analysis by reducing the size of the resulting data matrix. Methods to select uninformative features will typically include measures of variation such as standard deviation or interquartile range.

**REPORT:** Method for removal of uninformative features, including parameters, if used.

Removal of features present in process blanks (optional): It is highly recommended to analyse process blank samples during the analytical batch (see section 2). Features detected in these process blanks are thought to result from the solvents or plasticware rather than the biological sample and therefore can be considered for removal from the final data set. However, this can also be the result of carry-over in the injection system. This should be checked before features are removed.

**REPORT:** Method and parameters for process blank feature removal, if used.

Removal of features from dosed substances (optional): In toxicology studies, in which the biological system is deliberately exposed to an exogenous chemical agent, it is common to observe the dosed parent substance and/or its biotransformation products in the resulting data. For the purposes of analysing the endogenous metabolic effect of the exposure, it is important that these signals are removed from the data set. (Note that these signals would be left in the data set in cases where the objective is to analyse the metabolism of the dosed substance itself). Identification of the relevant variables to remove will typically involve comparison to control spectra from control non-dosed animals and standard substance spectra and/or literature. Typically variables identified for removal will simply be deleted from the data set.

**REPORT:** Method used to identify and remove features from dosed substances.

### Filtering of samples

For the same reasons as introduced in Section 9.4, the analytical measurement procedure may result in significant numbers of missing variables across samples. If multiple variables are missing for a particular sample, then that sample should be considered for removal. Often a threshold of 50% is used, whereby if a sample has >50% missing values then it is removed.

**REPORT**: Method for removal of samples, including threshold, if used.

## Supplementary Note 10. Statistical analysis

This section details general considerations for statistical analysis of the 2-dimensional data table produced by the preceding steps in the workflow (see Supplementary Figure 2). The statistical analysis plan will depend heavily on the objective and design of the study. For example, a single-treatment/control study to discover a chemical’s MoA requires a two-group analysis, while a benchmark dosing experiment requires an approach to dose-response modelling. Best practices for the four scenarios for regulatory application of metabolomics (Figure 2, main paper) have not yet been developed, hence these more complex designs and objectives are not considered here. However, some aspects of best practice are generic and applicable to all study designs.

At all steps of the statistical analysis, the software and parameters used must be reported. Open source software is preferable. If in-house scripts are used, deposition in a public repository (e.g. GitHub) is recommended.

**REPORT:** Software source, version and parameters used.

### Intrastudy QC analysis

A global overview of the data should be conducted to demonstrate that there are no remaining significant drifts/changes in intrastudy QC samples. This indicates that pre- and post-processing steps have been completed successfully and that the data is ready for further statistical analysis.

Univariate analysis: Each variable (i.e. peak or metabolic feature) should be analysed one at a time. An assessment should be made of the variance in intensity between the intrastudy QCs. The recommended default is by calculation of RSD (also known as CV).

**REPORT**: Distribution and median of RSD of variable intensities across all intrastudy QC samples. If another method is used, report the statistical approach used, including objective and parameters.

Multivariate analysis: A global principal component analysis (PCA) is recommended to demonstrate visually that the intrastudy QC samples a) have a lower variation than the study samples, and b) that there is little remaining systematic structure (e.g. run order drift) in the intrastudy QCs. If using PCA, all components explaining up to at least 50% of the total variance should be examined. Other multivariate techniques can be used (e.g. hierarchical clustering).

**REPORT:** PCA scores, loadings, variance explained and cross-validation statistics (e.g. Q^2^ goodness of fit for test samples) from a global analysis of all the biological and intrastudy QC samples.

### Biological sample outlier identification and removal

It is important to identify potential outlying biological samples and to remove if necessary before applying statistical analysis. Reasons for removing each outlier must be clearly explained in terms of biological, analytical or data analytical aspects; e.g. sample removed due to limited volume, contamination was detected, results of an independent assay indicating abnormality etc.

A multivariate method for outlier detection is recommended, e.g. PCA using Hotellings T^2^ on the scores and/or F-tests on the residuals.

**REPORT**: Multivariate method used, software and version; list of outlying samples removed with justification for each.

Univariate outlier analysis (optional). For example flagging outlying values for each variable as missing data. Care should be taken to consider the proportion of missing values resulting from this approach, potentially removing variables with too many missing values (see section 9.1).

**REPORT:** Univariate method used; summary of number of values excluded with justification.

### Normality testing, scaling and/or transformations

These are applied to each metabolite feature and are particularly important for multivariate analysis. They are typically performed to allow all features to contribute more evenly to a model, or to bring distributions closer to normality. Normality testing is of particular importance for selecting the appropriate statistical approach to use. Types of scaling include unit variance, Pareto, log, generalised log, range, level and no scaling. The appropriate type will depend on the nature of the data. For example, unit variance should not be applied where many variables report only noise. Similarly, generalised log is usually the best option for log scaling where zeros or negative numbers are present ^55^. Results should be interpreted in the light of the scaling used.

**REPORT:** Type of normality testing, scaling or transformation used and parameter (if relevant).

### Univariate analysis

Many different types of univariate statistical models and tests can be used depending on the experimental design. Each variable is modelled separately. For example, a simple t-test may be used to differentiate two groups (e.g. control vs. chemical exposed), or linear regression might be used to examine a relationship to a continuous outcome (e.g. liver mass).

**REPORT:** Univariate test used, software, version and (where relevant) parameters.

**REPORT:** Both the effect size (e.g. fold change) and statistical significance (p-value) of the result.

**REPORT:** Confidence intervals on all estimated parameters (optional).

Multiple testing corrections: When many variables are tested, as is always the case for untargeted metabolomics and often for targeted metabolite analysis, the likelihood of false positives is increased. In these circumstances, best practice is to adjust the significance level using a multiple hypothesis testing approach, such as those controlling the family wise error rate (FWER, e.g. Bonferroni correction) or false discovery rate (FDR, e.g. Benjamini & Hochberg). Additional reporting is then needed:

**REPORT:** Multiple testing approach, corrected p-values (q-values).

### Multivariate analysis - unsupervised

Many different types of unsupervised multivariate analysis can be applied. The goal of the unsupervised analysis is to provide an overview of the data to explore structure such as the major sources of variance, clustering or trends. This structure may result from, but not limited to, the following: the biological effects being studied (e.g. dose effect), uncontrolled natural biological variation, or residual variance in the analytical procedure. Best practice will include applying PCA to evaluate the data for such sources. Appropriate centering (e.g. using the mean of each variable) and scaling (technique dependent) should be used prior to PCA (see 10.3 above). All models must be validated (see 10.7 below).

**REPORT:** Multivariate method used, software, version and parameters.

**REPORT**: Output of the multivariate method (e.g. PCA scores, loadings, proportion of variance explained).

### Multivariate analysis - supervised

Supervised methods (usually classification or regression methods) are commonly used to focus the analysis on specific questions, e.g. whether the metabolic data can classify a sample into a chemical MoA, and to find which metabolic variables are most responsible for this classification. These methods are able to model data in cases where the treatment effect is small compared to other sources of variation. Many methods exist, but the chosen method should exhibit an ability to handle data with a) many variables, b) high degree of correlation between variables, c) high levels of noise, and d) missing data (if any). Recommended methods include partial least squares (PLS) or Orthogonal PLS (OPLS) regression when the outcome is continuous, or the equivalent discriminant analysis (PLS-DA and OPLS-DA) when the outcome is discrete (e.g. classification). Appropriate centering (e.g. using the mean of each variable) and scaling (technique dependent) should be used (see 10.3 above). All models must be validated (see 10.7 below).

**REPORT:** Multivariate method used, software, version and parameters.

**REPORT**: Output of the multivariate method (e.g. PLS scores, loadings/weights, regression coefficients, proportion of variance in outcome explained by the model).

### Multivariate analysis - validation

All models, both supervised and unsupervised, must be statistically validated to show that they are robust and predictive. This should be done by either a) separating the metabolomics data into independent training (typically a maximum of 70% of dataset) and test sets (remaining 30% minimum of dataset), or b) internal cross-validation. In both cases, summary statistics such as Q2 or misclassification rate should be calculated. Model complexity (e.g. number of principal components) should be chosen based on predictivity of the model. It is not recommended to use non-resampling criteria (e.g. Akaike Information Criterion AIC or Bayesian Information Criterion BIC) as they tend to result in overfitting with metabolomics data.

**REPORT:** Method used for model validation; validation statistics (Q2, error rate, etc.).

### Multivariate analysis - variable importance for feature selection

One of the main objectives of multivariate analysis in metabolomics studies is usually to determine which variables (e.g. metabolites) are important in the observed effect, e.g. those which show differential regulation between a chemical treatment and control. Many approaches are based on assessing the weight of each variable in the developed model. Best practice will include methods that are able to assess the statistical significance of these weights. For example, bootstrap procedures may be used to estimate confidence intervals on PCA loadings, allowing selection of variables where confidence intervals do not contain zero. Methods such as Variable Importance in Projection (VIP) or S-plot are able to rank variables by importance in PLS models. They can be used as long as a statistically sound approach to determining the significance threshold is used (e.g. bootstrap re-sampling).

**REPORT:** Method for variable importance/selection, including determination of significance threshold.

**REPORT**: Features selected by the method, feature importance (e.g. VIP value), feature significance (e.g. p-value, if available).

### Statistical analysis specific to regulatory toxicology scenarios

While the generic statistical approaches described above are usually applicable, studies in regulatory toxicology will require analyses tailored to the specific experimental design and question being asked. In most cases, the study will have a multiple dose and/or time point design, and this will need to be taken into account, e.g. multiple doses for BMD modelling, although no best practice for metabolomics-based BMD yet exists. Here we give some (non-exhaustive) guidance aligned with each of the four use case scenarios.

Deriving points of departure via benchmark dosing: Here, several doses are used to determine a point of departure from the control response and thus an estimate of the safe dose. A key decision is the definition of the benchmark response, i.e. the maximum acceptable additional risk beyond control (e.g. 10%) ^56^. Once this is decided, methods typically fit dose-response models to each metabolic variable (e.g., polynomial, Hill, exponential, etc.). An important consideration is selection of the best model type, ensuring that the response profile is adequately captured without overfitting the model. Finally, the benchmark dose and its lower confidence bound must be estimated and reported. We recommend the approach used by BMDExpress 2 ^57^ for transcriptomics as an example of best practice ^56^, which can handle multiple molecular variables in a single batch and hence could be applied in metabolomics.

**REPORT:** Definition of benchmark response; method, software, version and parameters used for modelling.

**REPORT:** Output of the approach including, for each metabolite reported, the type of dose-response model, model fit statistics, benchmark dose and lower confidence bound for the benchmark dose.

Mode of action discovery: In this scenario, metabolomics is used to discover metabolic key events, biomarkers and potentially pathways associated with the mode of action of the chemical. The experimental design will typically incorporate multiple doses and time points, and the determination of the sequence of events is of particular interest. A variety of statistical approaches may be used to ascertain which metabolic variables respond to the dose, at what times, and how these can be grouped into biologically meaningful sets (“pathways”). For example, univariate dose-response modelling might be applied to determine the sequence of metabolite responses. Metabolites might then be combined using databases/knowledgebases of predetermined metabolic pathways to investigate the biological processes involved in the response. Alternatively, multivariate methods could be applied to discover patterns of response coordinated across multiple metabolites, which could be analysed further according to pathways. Pathway analyses might also incorporate overrepresentation analysis ^58^, although a number of alternative approaches are available. Caution should be applied when attempting to infer causality from a set of time series metabolite measurements, unless the experimental design specifically addresses this (e.g. targeted inhibition of an enzyme).

**REPORT:** Metabolic biomarker/key event discovery approach (e.g. dose-response model), methods, software, version and parameters used; see sections above on uni- and multivariate analyses for further guidance.

**REPORT:** Pathway approach (if used), methods, software, version and parameters.

**REPORT:** Output of the approach (e.g. biomarkers, key events, pathways / mode of action.)

Chemical grouping for read-across: In this use case, the objective is to compare the metabolic responses of a series of chemicals (one or more test chemicals together with one or more reference chemicals with known MoA / toxicity), and then to group those chemicals according to the similarity (or not) of those metabolic responses. Then, within a group of chemicals, pre-existing toxicity data can be read-across from the reference set of chemical(s) to the test chemical(s). Hence, in chemical grouping, the emphasis is on the extent to which the test chemical’s metabolic phenotype matches that of the reference set, rather than attempting to determine the MoA. In this case, the appropriate statistical tools are pattern matching methods, of which a wide variety can be used. For example, a comparison of the response of each metabolite (e.g. up / down) to a pattern determined for a reference set with known MoA may allow classification of the test chemical according to predetermined confidence thresholds ^59^. A variety of other pattern matching and classification tools for chemical grouping are available ^60^. Whatever method is used, its accuracy on chemicals of known toxicity must be demonstrated, and care must be taken to avoid overfitting and bias. For each toxicological database with sufficient metabolomics data, it is recommended to perform a sensitivity analysis to determine the actual false discovery rate of the technology and procedure used as suggested by ^61^.

**REPORT:** Pattern matching / classification method used, software, version and parameters.

**REPORT:** Output of the chemical grouping (e.g. most similar compounds in reference set, similarity values, etc).

Cross-species extrapolation: The aim of this type of study is to determine if similar toxicological mechanisms are operating in different species under the same or similar chemical challenge. Thus, the expected outcomes combine some or all of those from the first three scenarios – benchmark doses, MoA and response similarity pattern matching. Hence the statistical methods and reporting requirements will draw on those used in the other three scenarios. In addition to similarities, there is an emphasis on investigating differences between species in how they respond to the chemical challenge. Other key points to take into account are the presence/absence of common metabolites, enzymes and pathways, differing response dynamics and changes in chemical potency across different species.

**REPORT:** Depending on the scenario, report as indicated above (e.g., benchmark dosing, MoA discovery and/or response similarity matching for chemical grouping).

## Supplementary Note 11. Metabolite identification

The analytical and informatic procedures for metabolite annotation and identification, together with their minimal reporting standards, are essential for allowing a critical appraisal and interpretation of results from a metabolomics study. Definitions for levels of confidence in metabolite identification were set out by the Metabolomics Standards Initiative (MSI) in 2007 ^62^ alongside various other proposals over several years ^63–67^, as summarized here ^68^. In addition, the levels of confidence in metabolite identification required for several use cases of metabolomics in regulatory toxicology have recently been discussed ^69^.

Level 1: *Identified metabolites* - for a feature to be ‘identified’ requires that 2 or more orthogonal properties of an authentic chemical standard are compared to the experimental data, both in the same laboratory and using the same method of data acquisition. Examples of such properties include retention time, accurate mass, fragmentation pattern, and/or NMR chemical shift.

Level 2: *Putatively annotated compounds* - this does not require comparison of acquired data to authentic chemical standards within the same laboratory. Instead, comparison to defined metabolite libraries (for example, NIST) is sufficient for *putative annotation*

Level 3: *Putatively characterised compound classes* - as for level 2, but where a specific match cannot be made. For example, hexoses share similar (and some cases identical) retention times and fragmentation patterns. Therefore, without an in house authentic standard (and potentially additional validation), the metabolite may only be annotated as a ‘hexose’, rather than ‘glucose’, ‘mannose’, ‘galactose’, ‘talose’, etc.

Level 4: *unknown compounds* - by definition, unknown compounds are those that fall outside the boundaries of the identification and annotation rules above.

These standards have continued to be revised, in part because their practical implementation has revealed some inadequacies and in part due to the greater capabilities of analytical instrumentation (in particular high resolution, high mass accuracy mass spectrometry) for metabolite identification ^65,66^. The role of updating and addressing the current and future requirements for reporting confidence in the levels of metabolite identification now resides with the Metabolomics Society’s Metabolite Identification Task Group. This group aims to harmonise various initiatives across the metabolomics community and to derive agreed standards for metabolite identification.

**REPORT:** Level of identification, common name, structural code (e.g. InChI or SMILES), metabolite standard/library (as appropriate).

## Supplementary Note 12. Management of data and metadata from regulatory metabolomics studies

No ‘best practice’ yet exists for the management of metadata and experimental data from metabolomics studies within regulatory toxicology. Yet significant opportunities exist to, first, benefit from the value of well curated (metabol)omics data in chemical risk assessment and, secondly, benefit from developments in related communities such as from academic metabolomics research ^70,71^. Consequently this section is structured differently to parts 1-11. It starts with highlighting some of the benefits that could arise from a new, more open approach to data sharing, then outlines the current approaches to data management both in the academic metabolomics community and in regulatory toxicology, and finishes with a proposed ‘best practice’ for data management and reporting. What is clear is that improved solutions are needed for managing data from metabolomics studies in regulatory toxicology.

### Why share data?

Publicly funded science has undergone a transformation in the last decade with a massive drive towards ‘open access’, i.e. the sharing of standardised and well curated metadata and experimental data, including metabolomics data ^71–74^. In 2016, FAIR (Findable, Accessible, Interoperable, and Re-usable) Data Principles ^75^ were published by a diverse set of stakeholders representing academia, funding agencies, scholarly publishers, and industry to act as a guideline for those intent on enhancing the reusability of their data. FAIR Data Principles emphasise enhancing the ability of machines to automatically find and use metadata and data. The benefits of sharing data include supporting discovery and innovation through interrogation of larger datasets, re-using existing data instead of repeating experimentation and measurement, gaining more credit for generating datasets through their reuse, and using shared data to build and validate new software tools. Yet, are any of the established benefits of data sharing of value to regulatory toxicology? The answer is of course yes, for both human health and environment, in the facilitation of better, more efficient, and well-informed science. This can be achieved by maximising use of existing (and new) data to build models for MoA prediction (Scenario 2, above), for chemical grouping and read-across (Scenario 3, above), and to reduce animal testing by avoiding repeated experimentation.

While there are considerable complexities associated with data generated for the purposes of regulatory toxicology versus that generated through publicly funded academic research (for example, within Europe, the toxicology data is generated and owned by industry), the benefits to industry of standardised data formats and data sharing are increasingly being realised. For example, the CDISC consortium’s (<www.cdisc.org>) mission ‘is to develop and support global, platform-independent data standards that enable information system interoperability to improve medical research and related areas of healthcare’. The consortium consists of over 450 member organisations, mostly from industry. It has released a Pharmacogenomics/Genetics domain model (CDISC PGx) to describe standards for the organisation, structure and format of gene-related datasets submitted as part of a product application to a regulatory agency. As a second example, the eTRANSAFE consortium (Enhancing TRANslational SAFEty Assessment through Integrative Knowledge Management; <http://etransafe.eu>) - with multiple industry partners - is developing an integrative data infrastructure and computational tools to improve the feasibility and reliability of translational safety assessment during drug development. The regulatory toxicology community could benefit substantially from the lessons learned by these existing consortia. In addition, earlier efforts in the field of transcriptomics can also help to identify the best path forward for metabolomics, e.g. the development of systems such as ArrayTrack ^76,77^, initial attempts at introducing key concepts around data management in regulatory transcriptomics ^78^, and identifying the core importance of metadata syntax, formats for raw data, and controlled vocabularies/semantic support. To aid the reader, semantic knowledge and information are used to classify content so that the knowledge it contains may be immediately retrieved, adding meaning to components of structured descriptions to help identify and interpret them unambiguously. Controlled vocabulary and ontology are arrangements of words and phrases used to organise knowledge, to index content or retrieve content. Ontology encompasses a representation, formal naming, definition, properties and relations between the concepts, data and entities.

### Current approaches to data management

Perhaps not surprisingly, the academic metabolomics community has already addressed their most immediate needs in data management and, independently, the regulatory toxicology community has well-developed solutions for traditional regulatory assays. Using Europe as an example, the EMBL-EBI and ELIXIR have significant expertise and capability in ‘omics data and metadata standards ^79,80^, reporting standards, and public databases and repositories ^81^. Examples include some of the foremost ‘omics databases and repositories in Europe including ArrayExpress (transcriptomics data repository ^82^), PRIDE (proteomics data repository ^83^ and MetaboLights (metabolomics data repository ^72^). Similarly, EMBL-EBI has significant additional expertise and capability in public chemical libraries, for example ChEBI ^84^ and ChEMBL ^85^, as well as other resources ^86,87^. Yet regulatory toxicology has not substantially benefited from these existing resources as yet.

Conversely, ECHA develops and maintains (under OECD governance) the IUCLID6 database that is focused on traditional regulatory toxicology data associated with legal frameworks such as REACH ^88^. This resource uses OECD Harmonised Templates (OHTs), which are standard data formats for reporting information derived from the risk assessment of chemicals (<https://www.oecd.org/ehs/templates>). Yet IUCLID6 and OHTs currently does not have capability to handle ‘omics data types. What has yet to happen (other than in a small number of commercial activities) is significant cross talk between the academic metabolomics and regulatory toxicology communities to discuss and advance a framework to manage, analyse and potentially share metabolomics data derived from regulatory toxicology studies. We recommend that this much needed cross talk occurs as part of the new OECD Metabolomics Reporting Framework project, considering the ideas presented below.

#### Metadata framework

For structuring the elements of experimental metadata, **ISA** ('Investigation' (the project context), 'Study' (a unit of research) and 'Assay' (analytical measurement)) ^89^ and **mwTab** ^90^ (which can be converted to ISA) provide two valuable, fit-for-purpose resources. Both are used in the principal international repositories for metabolomics data (EMBL-EBI MetaboLights and NIH Metabolomics Workbench ^90^), and could be considered for reuse in regulatory applications of metabolomics (Supplementary Table 3).

#### Raw data file formats

Within the metabolomics and wider research communities, major pushes towards open raw data file formats has been achieved, e.g. mzML ^91^ and mzTab ^92^ for mass spectrometry measurements, nmrML for NMR spectroscopy ^93^, qcML for quality control metrics in mass spectrometry ^94^, etc. In addition to raw data file formats, code libraries are now available to support them, facilitating their uptake and adoption as the knowledge of their existence spreads (Supplementary Table 3).

#### Controlled vocabulary and ontologies

Considerable progress has also been made in the field of semantics (controlled vocabularies and ontologies), which are now developed much like software packages, with modules that can be reused, augmented and contributed to, thus providing significant productivity gains (Supplementary Table 3). Resources from the OBOfoundry ^95^ are particularly relevant and power many model organism databases (e.g. RGD, MGI, Zebrafish, Flybase). Not only have the semantic resources grown as a set of interoperable tools, the software infrastructure for supporting the development, maintenance and release of controlled vocabularies has progressed tremendously, backed by extensive documentation (OBOFoundry.github.io, <http://robot.obolibrary.org>). These resources provide resolvable URI for annotation terms. As such, they provide an essential component to realising dataset FAIRification. To address the semantic needs in the application of metabolomics to regulatory toxicology, building on the Metabolomics Standards Initiative Ontology (MSIO) (<https://github.com/MSI-Metabolomics-Standards-Initiative/MSIO>), an application ontology aggregating key semantic descriptors from several resources, covering instrument descriptions and settings, statistical methods, study design, etc.) could be an effective strategy. Of particular relevance is its coverage of terminology for the informative and consistent description of QA/QC standards to help ensure metabolomics studies are conducted at a suitably high quality for regulatory applications.

*Supplementary Table 3. Overview of annotation guidelines, data formats and controlled terminologies (ontologies) relevant to the reporting of metabolomics studies. Standards presented in this table are linked to records available from the FAIRsharing repository. The first three columns define the subject of the data standards; the fourth column lists the guidelines (i.e. minimal checklists of requirements, which are not always fully and strictly formalized); the fifth column identifies syntaxes and data format specifications; the sixth and seventh columns identify semantic resources (i.e. provide structured controlled terminologies, sometimes interoperable and modular) for consumption by formal syntax in fifth column; the eighth column provides source of metabolic identities.*

| **Domain (relevant to each section of this paper)** | **Assay name** | **Technology** | **Reporting guideline** | **Standard format [primary data]** | **Specific semantic / terminology** | **Integrated semantic / terminology** | **Molecular identity declared in targeted studies** |
| --- | --- | --- | --- | --- | --- | --- | --- |
| Experimental Design Description and MetaData (1) | n/a | n/a | CIMR^^[[1]](#footnote-1)^^ | ISA^^[[2]](#footnote-2)^^ | OBI^^[[3]](#footnote-3)^^ | MSIO^^[[4]](#footnote-4)^^ | n/a |
|  | n/a | n/a | CIMR | ISA | STATO^^[[5]](#footnote-5)^^ | MSIO | n/a |
|  | n/a | n/a | CIMR | mwTab^^[[6]](#footnote-6)^^ | none supported (free text only) | none supported (free text only) | n/a |
|  | n/a | n/a | n/a | CDISC SEND^^[[7]](#footnote-7)^^ | CDISC Terminology^^[[8]](#footnote-8)^^ | CDISC Terminology | n/a |
| Quality Control (2) | n/a | n/a | n/a | QC-ML^^[[9]](#footnote-9)^^ | QCCv^^[[10]](#footnote-10)^^ | n/a | n/a |
| Quality Control (2) | n/a | n/a | n/a | ISA | STATO | MSIO | n/a |
| Data Acquisition Modality (4) | Untargeted metabolomics | NMR spectroscopy | CIMR | nmrML^^[[11]](#footnote-11)^^ | nmrCV^^[[12]](#footnote-12)^^ | MSIO | n/a |
| Data Acquisition Modality (5,6,7) | Untargeted metabolomics | mass spectrometry | CIMR | mzML^^[[13]](#footnote-13)^^ | PSI-MS^^[[14]](#footnote-14)^^ | MSIO | n/a |
| Data Acquisition Modality (5,6,7) | Untargeted metabolomics | mass spectrometry | CIMR | mzTab^^[[15]](#footnote-15)^^ | PSI-MS | MSIO | n/a |
| Data Acquisition Modality (8) | Targeted metabolite analysis | mass spectrometry | CIMR | mzML | PSI-MS | MSIO | (see metabolite list) |
| Data Acquisition Modality (8) | Targeted metabolite analysis | mass spectrometry | CIMR | mzTab | PSI-MS | MSIO | (see metabolite list) |
| Metabolite List (for targeted metabolite analysis; 8) | Targeted metabolite analysis | NMR spectroscopy, mass spectrometry |  | json datapackage^^[[16]](#footnote-16)^^ | miscellaneous | MSIO | ChEBI; ChEMBL;InChI |
| Data Analysis Workflow (9,10) | n/a | n/a | n/a | CWL^^[[17]](#footnote-17)^^ | EDAM^^[[18]](#footnote-18)^^ | not known | n/a |
| Data Analysis Workflow (9,10) | n/a | n/a | n/a | BCO | not known | not known | n/a |
| Data Analysis Workflow (9,10) | n/a | n/a | n/a | WDL | not known | not known | n/a |
| Analysis Results Matrix (9,10) | n/a | NMR spectroscopy, mass spectrometry | n/a | json datapackage | STATO |  | n/a |
| Analysis Results Matrix (9,10) | n/a | n/a |  | OECD harmonised reporting template^^[[19]](#footnote-19)^^ (XML for developers) | not known | not known | n/a |
| Chemical Identity (11) | n/a | NMR spectroscopy, mass spectrometry | n/a | InChI^^[[20]](#footnote-20)^^ | n/a | n/a | n/a |

### Recommended reporting of data and metadata

The regulatory toxicology community in partnership with ‘omics experts need to implement a strategy for (metabol)omics data management, to ensure these technologies can most effectively contribute to hazard assessment. The community is in a strong position to capitalise on existing experience and practice to advance these standardisation efforts apace. Given the need to work with a new data type, arguably this is a good time to review policies on restricted access to data, and create the opportunity for the data owners (typically industry) to benefit not only from generating and utilising their own metabolomics data, but to initiate wider data sharing and its reuse. Using Europe as an example, Figure 5 (main paper) proposes a (metabol)omics data management strategy that would (1) allow compliance standards to be checked by ECHA (through the extension of an OHT template to capture critical information describing the data source, data quality, results and interpretation from the metabolomics experiment), and (2) report the full metabolomics data and metadata to an access-controlled specialised (metabol)omics data repository. Here, we term path 1 the ‘Regulatory compliance path’, and path 2 the ‘Complete data path’ for data submission. The access control for the complete data path could be set to ‘private data’ (i.e. available only to the industry owner and ECHA), or ‘public data’ (i.e. open access, for shared reuse and data mining). A metabolomics study would involve data submission to both paths, not only one.

Raw data and metadata file formats:

**REPORT (for proposed ‘regulatory compliance path’):** File format should utilise an extended OECD Harmonised Reporting Template (e.g. based on OHT 201 Intermediate Effects).

**REPORT (for proposed ‘complete data path’):** Study metadata should be described using the ISA model; metabolomics data should be stored using open data formats (e.g. mzML, nmrML).

Metabolomics data and metadata deposition:

**REPORT (for proposed ‘regulatory compliance path’)**: Submission of sufficient data/metadata for a compliance check should occur to the established, regional, access-controlled data repository for regulatory toxicology (e.g. IUCLID6 in Europe and increasingly in other countries).

**REPORT (for proposed ‘complete data path’):** Submission of a complete package of data/metadata to an established, regional, specialised data repository for metabolomics studies, either the European-based MetaboLights (<https://www.ebi.ac.uk/metabolights>) or US-based Metabolomics Workbench (<http://www.metabolomicsworkbench.org>), including accession number and conforming to FAIR Data Principles (note the data repository should have an access-controlled area as ‘Accessibility’ in the FAIR Data Principles does not equate to ‘open’, rather it forces qualification and declarations of conditions of access).

*Supplementary Table 4. Summary of minimal best practice and reporting criteria for untargeted metabolomics and targeted metabolite analysis in regulatory toxicology.*

| **Type of metabolomics analysis** | **Regulatory toxicology scenarios** | **Minimal use of QC samples** | **Minimal use of internal standards** | **Minimal level of quantification** | **Minimal level of metabolite annotation / identification** |
| --- | --- | --- | --- | --- | --- |
| **Untargeted metabolomics** | · MoA/KE discovery  Deriving point of departure  · Chemical grouping and read-across  · Cross species extrapolation | · System suitability QC  Intrastudy QC  · Process blank QC | Not needed. Although an internal standard is routinely used in NMR metabolomics | Relative quantification | Any MSI level allowed for each metabolite (currently levels 1-4; under refinement by MSI); level of confidence must be reported |
| **Targeted metabolite analysis**  (of 1 to few 100 metabolites or biomarkers; termed targeted regulatory qualification in FDA literature). | · Deriving point of departure  · Grouping & read-across  · Cross species extrapolation | · System suitability QC  · Process blank QC | Required. Ideally a labelled internal standard for each quantified metabolite. Minimally need one internal standard for each metabolite class (where it has been proven that standard is sufficiently representative of class) | Ideally absolute quantitation (one internal standard per metabolite), as this allows interstudy and interlab comparisons. Minimally semi-quantitative (for case where one internal standard per metabolite class) | Ideally MSI level 1 (definitive identification confirmed with authentic standard). However, since not all standards are commercially available, MSI level 2 can be accepted |

**Supplementary References**

1. Ramirez, T. *et al.* Prediction of liver toxicity and mode of action using metabolomics in vitro in HepG2 cells. *Arch. Toxicol.* **92**, 893–906 (2018).

2. Cuykx, M., Claes, L., Rodrigues, R. M., Vanhaecke, T. & Covaci, A. Metabolomics profiling of steatosis progression in HepaRG® cells using sodium valproate. *Toxicol. Lett.* **286**, 22–30 (2018).

3. Kamp, H. *et al.* Reproducibility and robustness of metabolome analysis in rat plasma of 28-day repeated dose toxicity studies. *Toxicol. Lett.* **215**, 143–149 (2012).

4. Taylor, N. S., Gavin, A. & Viant, M. R. Metabolomics Discovers Early-Response Metabolic Biomarkers that Can Predict Chronic Reproductive Fitness in Individual Daphnia magna. *Metabolites* **8**, (2018).

5. Ebbels, T. M. D., Holmes, E., Lindon, J. C. & Nicholson, J. K. Evaluation of metabolic variation in normal rat strains from a statistical analysis of 1H NMR spectra of urine. *J. Pharm. Biomed. Anal.* **36**, 823–833 (2004).

6. Vaidyanathan, S. *et al.* A laser desorption ionisation mass spectrometry approach for high throughput metabolomics. *Metabolomics* **1**, 243–250 (2005).

7. Gavaghan McKee, C. L., Wilson, I. D. & Nicholson, J. K. Metabolic phenotyping of nude and normal (Alpk:ApfCD, C57BL10J) mice. *J. Proteome Res.* **5**, 378–384 (2006).

8. Espandiari, P. *et al.* Age-related differences in susceptibility to cisplatin-induced renal toxicity. *J. Appl. Toxicol.* **30**, 172–182 (2010).

9. Viant, M. R., Bundy, J. G., Pincetich, C. A., de Ropp, J. S. & Tjeerdema, R. S. NMR-derived developmental metabolic trajectories: an approach for visualizing the toxic actions of trichloroethylene during embryogenesis. *Metabolomics* **1**, 149–158 (2005).

10. Bollard, M. E. *et al.* Investigations into biochemical changes due to diurnal variation and estrus cycle in female rats using high-resolution (1)H NMR spectroscopy of urine and pattern recognition. *Anal. Biochem.* **295**, 194–202 (2001).

11. Stanley, E. G. *et al.* Sexual dimorphism in urinary metabolite profiles of Han Wistar rats revealed by nuclear-magnetic-resonance-based metabonomics. *Anal. Biochem.* **343**, 195–202 (2005).

12. Griffin, J. L. *et al.* Standard reporting requirements for biological samples in metabolomics experiments: mammalian/in vivo experiments. *Metabolomics* **3**, 179–188 (2007).

13. van der Werf, M. J. *et al.* Standard reporting requirements for biological samples in metabolomics experiments: microbial and in vitro biology experiments. *Metabolomics* **3**, 189–194 (2007).

14. Schneider, K. *et al.* ‘ToxRTool’, a new tool to assess the reliability of toxicological data. *Toxicol. Lett.* **189**, 138–144 (2009).

15. Southam, A. D. *et al.* Metabolomics reveals target and off-target toxicities of a model organophosphate pesticide to roach (Rutilus rutilus): implications for biomonitoring. *Environ. Sci. Technol.* **45**, 3759–3767 (2011).

16. OECD. *Test No. 417: Toxicokinetics*. (OECD, 2010).

17. Li, H.-H. *et al.* Development of a toxicogenomics signature for genotoxicity using a dose-optimization and informatics strategy in human cells. *Environ. Mol. Mutagen.* **56**, 505–519 (2015).

18. Soanes, K. H. *et al.* Molecular characterization of zebrafish embryogenesis via DNA microarrays and multiplatform time course metabolomics studies. *J. Proteome Res.* **10**, 5102–5117 (2011).

19. An, P. N. T., Yamaguchi, M., Bamba, T. & Fukusaki, E. Metabolome analysis of Drosophila melanogaster during embryogenesis. *PLoS One* **9**, e99519 (2014).

20. Dunn, W. B. *et al.* Quality assurance and quality control processes: summary of a metabolomics community questionnaire. *Metabolomics* **13**, 50 (2017).

21. Kauffmann, H.-M. *et al.* Framework for the quality assurance of ’omics technologies considering GLP requirements. *Regul. Toxicol. Pharmacol.* **91 Suppl 1**, S27–S35 (2017).

22. Broadhurst, D. *et al.* Guidelines and considerations for the use of system suitability and quality control samples in mass spectrometry assays applied in untargeted clinical metabolomic studies. *Metabolomics* **14**, 72 (2018).

23. Gika, H. G., Theodoridis, G. A., Wingate, J. E. & Wilson, I. D. Within-day reproducibility of an HPLC-MS-based method for metabonomic analysis: application to human urine. *J. Proteome Res.* **6**, 3291–3303 (2007).

24. Sangster, T., Major, H., Plumb, R., Wilson, A. J. & Wilson, I. D. A pragmatic and readily implemented quality control strategy for HPLC-MS and GC-MS-based metabonomic analysis. *Analyst* **131**, 1075–1078 (2006).

25. Dunn, W. B. *et al.* Procedures for large-scale metabolic profiling of serum and plasma using gas chromatography and liquid chromatography coupled to mass spectrometry. *Nat. Protoc.* **6**, 1060–1083 (2011).

26. Beger, R. D. *et al.* Towards quality assurance and quality control in untargeted metabolomics studies. *Metabolomics* **15**, 4 (2019).

27. Beckonert, O. *et al.* Metabolic profiling, metabolomic and metabonomic procedures for NMR spectroscopy of urine, plasma, serum and tissue extracts. *Nat. Protoc.* **2**, 2692–2703 (2007).

28. Bordag, N. *et al.* Fast Sampling of Adherent Cell Cultures for Optimal Metabolomics Results. *Metabolomics:Open Access* **6**, 1–12 (2016).

29. Martano, G. *et al.* Fast sampling method for mammalian cell metabolic analyses using liquid chromatography-mass spectrometry. *Nat. Protoc.* **10**, 1–11 (2015).

30. Bennett, B. D., Yuan, J., Kimball, E. H. & Rabinowitz, J. D. Absolute quantitation of intracellular metabolite concentrations by an isotope ratio-based approach. *Nat. Protoc.* **3**, 1299–1311 (2008).

31. Rabinowitz, J. D. & Kimball, E. Acidic acetonitrile for cellular metabolome extraction from Escherichia coli. *Anal. Chem.* **79**, 6167–6173 (2007).

32. Breier, M. *et al.* Targeted metabolomics identifies reliable and stable metabolites in human serum and plasma samples. *PLoS One* **9**, e89728 (2014).

33. Anton, G. *et al.* Pre-analytical sample quality: metabolite ratios as an intrinsic marker for prolonged room temperature exposure of serum samples. *PLoS One* **10**, e0121495 (2015).

34. Haid, M. *et al.* Long-Term Stability of Human Plasma Metabolites during Storage at −80 °C. *Journal of Proteome Research* **17**, 203–211 (2018).

35. Pinto, J. *et al.* Human plasma stability during handling and storage: impact on NMR metabolomics. *Analyst* **139**, 1168–1177 (2014).

36. Rotter, M. *et al.* Stability of targeted metabolite profiles of urine samples under different storage conditions. *Metabolomics* **13**, 4 (2017).

37. Torell, F. *et al.* The effects of thawing on the plasma metabolome: evaluating differences between thawed plasma and multi-organ samples. *Metabolomics* **13**, 66 (2017).

38. Petrick, L. *et al.* An untargeted metabolomics method for archived newborn dried blood spots in epidemiologic studies. *Metabolomics* **13**, (2017).

39. Drolet, J. *et al.* Integrated Metabolomics Assessment of Human Dried Blood Spots and Urine Strips. *Metabolites* **7**, (2017).

40. Wu, H., Southam, A. D., Hines, A. & Viant, M. R. High-throughput tissue extraction protocol for NMR- and MS-based metabolomics. *Anal. Biochem.* **372**, 204–212 (2008).

41. Tufi, S., Lamoree, M., de Boer, J. & Leonards, P. Simultaneous analysis of multiple neurotransmitters by hydrophilic interaction liquid chromatography coupled to tandem mass spectrometry. *J. Chromatogr. A* **1395**, 79–87 (2015).

42. Dona, A. C. *et al.* Precision high-throughput proton NMR spectroscopy of human urine, serum, and plasma for large-scale metabolic phenotyping. *Anal. Chem.* **86**, 9887–9894 (2014).

43. Ludwig, C. & Viant, M. R. Two-dimensional J-resolved NMR spectroscopy: review of a key methodology in the metabolomics toolbox. *Phytochem. Anal.* **21**, 22–32 (2010).

44. Want, E. J. *et al.* Global metabolic profiling of animal and human tissues via UPLC-MS. *Nat. Protoc.* **8**, 17–32 (2013).

45. Bonfiglio, R., King, R. C., Olah, T. V. & Merkle, K. The effects of sample preparation methods on the variability of the electrospray ionization response for model drug compounds. *Rapid Commun. Mass Spectrom.* **13**, 1175–1185 (1999).

46. Matuszewski, B. K., Constanzer, M. L. & Chavez-Eng, C. M. Strategies for the Assessment of Matrix Effect in Quantitative Bioanalytical Methods Based on HPLC−MS/MS. *Anal. Chem.* **75**, 3019–3030 (2003).

47. Ogawa, T., Okazawa, A. & Ohta, D. A protocol for GC–MS-based metabolomic analysis in mature seed of rice (_Oryza sativa_ L.). (2017). doi:10.1038/protex.2017.151

48. Hušek, P. *et al.* GC-MS Metabolomic Profiling of Protic Metabolites Following Heptafluorobutyl Chloroformate Mediated Dispersive Liquid Microextraction Sample Preparation Protocol. *Methods Mol. Biol.* **1738**, 159–181 (2018).

49. Seifar, R. M., Ten Pierick, A. & van Dam, P. T. N. Analysis of Metabolites from the Tricarboxylic Acid Cycle for Yeast and Bacteria Samples Using Gas Chromatography Mass Spectrometry. *Methods Mol. Biol.* **1730**, 277–282 (2018).

50. Southam, A. D., Weber, R. J. M., Engel, J., Jones, M. R. & Viant, M. R. A complete workflow for high-resolution spectral-stitching nanoelectrospray direct-infusion mass-spectrometry-based metabolomics and lipidomics. *Nat. Protoc.* **12**, 310–328 (2016).

51. Kirwan, J. A., Weber, R. J. M., Broadhurst, D. I. & Viant, M. R. Direct infusion mass spectrometry metabolomics dataset: a benchmark for data processing and quality control. *Sci Data* **1**, 140012 (2014).

52. Siskos, A. P. *et al.* Interlaboratory Reproducibility of a Targeted Metabolomics Platform for Analysis of Human Serum and Plasma. *Anal. Chem.* **89**, 656–665 (2017).

53. Hrydziuszko, O. & Viant, M. R. Missing values in mass spectrometry based metabolomics: an undervalued step in the data processing pipeline. *Metabolomics* **8**, 161–174 (2012).

54. Wei, R. *et al.* Missing Value Imputation Approach for Mass Spectrometry-based Metabolomics Data. *Sci. Rep.* **8**, 663 (2018).

55. Parsons, H. M., Ludwig, C., Günther, U. L. & Viant, M. R. Improved classification accuracy in 1- and 2-dimensional NMR metabolomics data using the variance stabilising generalised logarithm transformation. *BMC Bioinformatics* **8**, 234 (2007).

56. Yang, L., Allen, B. C. & Thomas, R. S. BMDExpress: a software tool for the benchmark dose analyses of genomic data. *BMC Genomics* **8**, 387 (2007).

57. Phillips, J. R. *et al.* BMDExpress 2: Enhanced transcriptomic dose-response analysis workflow. *Bioinformatics* (2018). doi:10.1093/bioinformatics/bty878

58. Xia, J. & Wishart, D. S. MSEA: a web-based tool to identify biologically meaningful patterns in quantitative metabolomic data. *Nucleic Acids Res.* **38**, W71–7 (2010).

59. van Ravenzwaay, B. *et al.* Metabolomics as read-across tool: A case study with phenoxy herbicides. *Regul. Toxicol. Pharmacol.* **81**, 288–304 (2016).

60. Ebbels, T. M. D. *et al.* Prediction and classification of drug toxicity using probabilistic modeling of temporal metabolic data: the consortium on metabonomic toxicology screening approach. *J. Proteome Res.* **6**, 4407–4422 (2007).

61. Ravenzwaay, B. V. *et al.* The development of a database for metabolomics - looking back on ten years of experience. *Int. J. Biotechnol.* **14**, 47–68 (2015).

62. Sumner, L. W. *et al.* Proposed minimum reporting standards for chemical analysis Chemical Analysis Working Group (CAWG) Metabolomics Standards Initiative (MSI). *Metabolomics* **3**, 211–221 (2007).

63. Dona, A. C. *et al.* A guide to the identification of metabolites in NMR-based metabonomics/metabolomics experiments. *Comput. Struct. Biotechnol. J.* **14**, 135–153 (2016).

64. Creek, D. J. *et al.* Metabolite identification: are you sure? And how do your peers gauge your confidence? *Metabolomics* **10**, 0 (2014).

65. Dunn, W. B. *et al.* Mass appeal: metabolite identification in mass spectrometry-focused untargeted metabolomics. *Metabolomics* **9**, 44–66 (2013).

66. Schymanski, E. L. *et al.* Identifying small molecules via high resolution mass spectrometry: communicating confidence. *Environ. Sci. Technol.* **48**, 2097–2098 (2014).

67. Blaženović, I., Kind, T., Ji, J. & Fiehn, O. Software Tools and Approaches for Compound Identification of LC-MS/MS Data in Metabolomics. *Metabolites* **8**, (2018).

68. Salek, R. M., Steinbeck, C., Viant, M. R., Goodacre, R. & Dunn, W. B. The role of reporting standards for metabolite annotation and identification in metabolomic studies. *Gigascience* **2**, 13 (2013).

69. Malinowska, J. M. & Viant, M. R. Confidence in metabolite identification dictates the applicability of metabolomics to regulatory toxicology. *Current Opinion in Toxicology* **16**, 32–38 (2019).

70. Haug, K., Salek, R. M. & Steinbeck, C. Global open data management in metabolomics. *Curr. Opin. Chem. Biol.* **36**, 58–63 (2017).

71. Rocca-Serra, P. *et al.* Data standards can boost metabolomics research, and if there is a will, there is a way. *Metabolomics* **12**, 14 (2015).

72. Haug, K. *et al.* MetaboLights—an open-access general-purpose repository for metabolomics studies and associated meta-data. *Nucleic Acids Res.* (2012). doi:10.1093/nar/gks1004

73. Website. Available at: https://doi.org/10.1007/s11306-012-0462-0. (Accessed: 12th September 2018)

74. Sud, M. *et al.* Metabolomics Workbench: An international repository for metabolomics data and metadata, metabolite standards, protocols, tutorials and training, and analysis tools. *Nucleic Acids Res.* **44**, D463–70 (2016).

75. Wilkinson, M. D. *et al.* The FAIR Guiding Principles for scientific data management and stewardship. *Sci Data* **3**, 160018 (2016).

76. Tong, W. *et al.* ArrayTrack--supporting toxicogenomic research at the U.S. Food and Drug Administration National Center for Toxicological Research. *Environ. Health Perspect.* **111**, 1819–1826 (2003).

77. Tong, W. *et al.* Development of public toxicogenomics software for microarray data management and analysis. *Mutat. Res.* **549**, 241–253 (2004).

78. Burgoon, L. D. Clearing the standards landscape: the semantics of terminology and their impact on toxicogenomics. *Toxicol. Sci.* **99**, 403–412 (2007).

79. van Rijswijk, M. *et al.* The future of metabolomics in ELIXIR. *F1000Res.* **6**, (2017).

80. Crosswell, L. C. & Thornton, J. M. 6.12 - EBI and ELIXIR. in *Comprehensive Biomedical Physics* (ed. Brahme, A.) 175–190 (Elsevier, 2014).

81. Cook, C. E. *et al.* The European Bioinformatics Institute in 2016: Data growth and integration. *Nucleic Acids Res.* **44**, D20–6 (2016).

82. Rustici, G. *et al.* ArrayExpress update--trends in database growth and links to data analysis tools. *Nucleic Acids Res.* **41**, D987–90 (2013).

83. Jones, P. *et al.* PRIDE: a public repository of protein and peptide identifications for the proteomics community. *Nucleic Acids Res.* **34**, D659–63 (2006).

84. Hastings, J. *et al.* ChEBI in 2016: Improved services and an expanding collection of metabolites. *Nucleic Acids Res.* **44**, D1214–9 (2016).

85. Bento, A. P. *et al.* The ChEMBL bioactivity database: an update. *Nucleic Acids Res.* **42**, D1083–90 (2014).

86. Kim, S. *et al.* PubChem Substance and Compound databases. *Nucleic Acids Res.* **44**, D1202–13 (2016).

87. Wishart, D. S. *et al.* HMDB 3.0--The Human Metabolome Database in 2013. *Nucleic Acids Res.* **41**, D801–7 (2013).

88. Führ, M. & Bizer, K. REACh as a paradigm shift in chemical policy--responsive regulation and behavioural models. *J. Clean. Prod.* **15**, 327–334 (2007).

89. Rocca-Serra, P. *et al.* ISA software suite: supporting standards-compliant experimental annotation and enabling curation at the community level. *Bioinformatics* **26**, 2354–2356 (2010).

90. Smelter, A. & Moseley, H. N. B. A Python library for FAIRer access and deposition to the Metabolomics Workbench Data Repository. *Metabolomics* **14**, 64 (2018).

91. Martens, L. *et al.* mzML—a Community Standard for Mass Spectrometry Data. *Mol. Cell. Proteomics* **10**, (2011).

92. Griss, J. *et al.* The mzTab data exchange format: communicating mass-spectrometry-based proteomics and metabolomics experimental results to a wider audience. *Mol. Cell. Proteomics* **13**, 2765–2775 (2014).

93. Schober, D. *et al.* nmrML: a community supported open data standard for the description, storage, and exchange of NMR data. *Anal. Chem.* (2017).

94. Bittremieux, W. *et al.* The Human Proteome Organization-Proteomics Standards Initiative Quality Control Working Group: Making Quality Control More Accessible for Biological Mass Spectrometry. *Anal. Chem.* **89**, 4474–4479 (2017).

95. Smith, B. *et al.* The OBO Foundry: coordinated evolution of ontologies to support biomedical data integration. *Nat. Biotechnol.* **25**, 1251–1255 (2007).

1. https://fairsharing.org/10.25504/fairsharing.exz30t [↑](#footnote-ref-1)
2. https://fairsharing.org/FAIRsharing.53gp75 [↑](#footnote-ref-2)
3. https://fairsharing.org/FAIRsharing.284e1z [↑](#footnote-ref-3)
4. https://github.com/MSI-Metabolomics-Standards-Initiative/MSIO [↑](#footnote-ref-4)
5. https://fairsharing.org/FAIRsharing.na5xp [↑](#footnote-ref-5)
6. https://fairsharing.org/bsg-s001215/ [↑](#footnote-ref-6)
7. https://fairsharing.org/FAIRsharing.7z842d [↑](#footnote-ref-7)
8. https://fairsharing.org/10.25504/FAIRsharing.szrmev [↑](#footnote-ref-8)
9. https://fairsharing.org/10.25504/fairsharing.dmhjcg [↑](#footnote-ref-9)
10. https://github.com/HUPO-PSI/qcML-development/blob/master/cv/v0_0_11/qc-cv.obo [↑](#footnote-ref-10)
11. https://fairsharing.org/10.25504/fairsharing.es03fk [↑](#footnote-ref-11)
12. https://fairsharing.org/FAIRsharing.xm7tkj [↑](#footnote-ref-12)
13. https://fairsharing.org/10.25504/fairsharing.26dmba [↑](#footnote-ref-13)
14. https://fairsharing.org/FAIRsharing.sxh2dp [↑](#footnote-ref-14)
15. https://fairsharing.org/FAIRsharing.c12tyk [↑](#footnote-ref-15)
16. https://frictionlessdata.io/specs/data-package/ [↑](#footnote-ref-16)
17. https://fairsharing.org/10.25504/fairsharing.8y5ayx [↑](#footnote-ref-17)
18. https://fairsharing.org/FAIRsharing.a6r7zs [↑](#footnote-ref-18)
19. https://www.oecd.org/ehs/templates/ALL%20XSD%20Files.zip [↑](#footnote-ref-19)
20. https://fairsharing.org/FAIRsharing.ddk9t9 [↑](#footnote-ref-20)
